# Platelet-, monocyte-derived and tissue factor-carrying circulating microparticles are related to acute myocardial infarction severity

**DOI:** 10.1371/journal.pone.0172558

**Published:** 2017-02-16

**Authors:** Gemma Chiva-Blanch, Kristian Laake, Peder Myhre, Vibeke Bratseth, Harald Arnesen, Svein Solheim, Lina Badimon, Ingebjørg Seljeflot

**Affiliations:** 1 Centre for Clinical Heart Research, Department of Cardiology, Oslo University Hospital Ullevål, Oslo, Norway; 2 Faculty of Medicine, University of Oslo, Oslo, Norway; 3 Cardiovascular Research Centre (CSIC-ICCC) and Biomedical Research Institute Sant Pau (IIB-Sant Pau), Barcelona, Spain; GERMANY

## Abstract

**Objective:**

Circulating microparticles (cMPs) are phospholipid-rich vesicles released from cells when activated or injured, and contribute to the formation of intracoronary thrombi. Tissue factor (TF, CD142) is the main trigger of fibrin formation and TF-carrying cMPs are considered one of the most procoagulant cMPs. Similar types of atherosclerotic lesions may lead to different types of AMI, although the mechanisms behind are unresolved. Therefore, we aimed to investigate the phenotype of cMPs found in plasma of ACS patients and its relation to AMI severity and thrombotic burden.

**Methods:**

In a cross-sectional study, two hundred patients aged 75±4 years were included in the study 2–8 weeks after suffering an AMI. Annexin V positive (AV^+^)-cMPs derived from blood and vascular cells were measured by flow cytometry. Plasma procoagulant activity (TF-PCA) was measured through a chromogenic assay.

**Results:**

STEMI patients (n = 75) showed higher levels of platelet-derived cMPs [CD61^+^/AV^+^, CD31^+^/AV^+^, CD42b^+^/AV^+^ and CD31^+^/CD42b^+^/AV^+^, *P* = 0.048, 0.038, 0.009 and 0.006, respectively], compared to NSTEMI patients (n = 125). Patients who suffered a heart failure during AMI (n = 17) had increased levels of platelet (CD61^+^)-and monocyte (CD14^+^)-derived cMPs carrying TF (CD142^+^) (*P*<0.0001 and 0.004, respectively). Additionally, NYHA class III (n = 23) patients showed higher levels of CD142^+^/AV^+^, CD14^+^/AV^+^ and CD14^+^/CD142^+^/AV^+^ cMPs than those in class I/II (*P* = 0.001, 0.015 and 0.014, respectively). The levels of these cMPs positively correlated with TF-PCA (r≥0.166, *P*≤0.027, all).

**Conclusions:**

Platelets and monocytes remain activated in AMI patients treated as per guidelines and release cMPs that discriminate AMI severity. Therefore, TF-MPs, and platelet- and monocyte-MPs may reflect thrombotic burden in AMI patients.

## Introduction

Acute myocardial infarction (AMI) is mainly triggered by a disruption of an atherosclerotic plaque in an epicardial coronary artery, which may result in activation of the clotting cascade, with subsequent thrombus formation and occlusion of the artery. Similar types of atherosclerotic lesions may lead to different types of AMI, ST-elevation myocardial infarction (STEMI) or non-STEMI (NSTEMI). The mechanisms behind are still unresolved, but important because of their clinical implications[1].

Circulating microparticles (cMPs) are small extracellular vesicles, released from circulating blood and vascular cells during damage or stress, or when undergoing apoptotic or necrotic process. cMPs have been related to the atherosclerotic process including chronic inflammation, and have also been related to intracoronary thrombosis and AMI[2–5]. cMPs expose phosphatidylserine (PS) in the outer surface layer as a consequence of their formation and release, and contain different molecules from their parental cells which may determine their capacities in promoting blood coagulation and cell activation[6].

Tissue factor (TF, CD142) is the cellular initiator of the extrinsic coagulation cascade and plays a key role in intravascular thrombus formation. Indeed, TF-carrying cMPs are considered the main reservoir of plasma TF activity[7]. Elevated levels of cMPs and TF-carrying cMPs have been observed in AMI patients compared to control subjects[5, 7–9] and in patients with unstable angina[10], but the involvement of cMPs in AMI types and severity, and its relation to middle-long term prognosis remains unclear.

Therefore, we aimed to investigate the phenotype of cMPs found in plasma 2–8 weeks after an AMI, and its relation to AMI severity, because we hypothesized that they may discriminate AMI severity and the procoagulant status at middle term.

## Materials and methods

### Patients

In this cross-sectional study, two hundred patients 70–82 years old, belonging to a subset of the OMEMI cohort[11], were included in the study at Oslo University Hospital (OUS Ullevål), Norway, 2 to 8 weeks after suffering an AMI, to limit the influence of the acute setting and index medication. Patients were recruited between November 2012 and December 2014. They were all treated as per guidelines recommendations. Demographic and clinical characteristics were obtained from all patients using a standardized report form at inclusion. The Regional Committee for Medical Research Ethics approved the study protocol that adheres to the principles outlined in the Declaration of Helsinki. Patients gave written informed consent before inclusion. The study is registered at Clinical-Trials.gov as NCT01841944.

Fasting venous blood was drawn in the morning between 08:00 and 10:30. Tubes containing 3.8% sodium citrate were used for MP and procoagulant activity (TF-PCA) analysis. Blood cells were removed by centrifugation (2500×g, 20min) at 4°C and plasma was immediately frozen and stored at -80°C until analyzed.

### Circulating microparticles characterization and quantification

The cMP fraction was isolated from plasma by a two-step high-speed centrifugation. Five hundred μL of frozen plasma aliquots were thawed on melting ice and centrifuged again at 2,500g 10 min at room temperature (RT). Then, 250 μL of plasma collected from the upper part of the vial was transferred to another vial and centrifuged at 20,000×g for 30 min at RT to pellet cMPs. The supernatants were discarded and the cMP enriched pellet was washed once with citrate-phosphate buffered saline (PBS) solution (citrate-PBS; 1.4 mmol/L phosphate, 154 mmol/L NaCl, 10.9 mmol/L trisodium citrate, pH 7.4) before a second equal centrifugation step was made. Finally, the remaining cMP pellets were resuspended in 100 μL citrate-PBS.

Triple-label flow cytometric analysis was performed as described before[12, 13] adapted to 96-well plates. Briefly, washed cMP suspensions were diluted in PBS buffer containing 2.5 mM CaCl_2_ (Annexin Binding Buffer, ABB). Thereafter, combinations of allophycocyanin (APC)-conjugated Annexin V (AV) with two specific monoclonal antibodies (mAb, Tables A and B in S1 Appendix) labeled with fluorescein isothiocyanate (FITC) and phycoerythrin (PE), or the isotype-matched control antibodies, were added.

Samples were incubated 20 min at RT in the dark and diluted with ABB before being immediately analyzed by flow cytometry, except for cMPs from smooth muscle cells (SMC). For these cMPs, 5 μL of the cMP suspension were incubated 20 min at RT in the dark with 5 μL AV-APC and 5 μL CD142-FITC (tissue factor, TF) in a final volume of 50 μL ABB. As previously described[14], cMPs were fixed with ABB/paraformaldehyde 2% during 30 min and centrifuged at 20,000×g for 30 min to pellet cMPs. After eliminating the supernatant, cMPs were permeabilized with ABB/saponin 0.1% 20 min at RT in the dark. After permeabilizing, 5 μL of smooth muscle actin (SMA)-α-PE were added to the cMPs suspension and incubated 20 min at RT in the dark and finally diluted with ABB prior to flow cytometer analyses. Samples were analyzed with the Auto Collect mode in 96-well plates on an AccuriC6 flow cytometer (BD, Accuri^®^ Cytometers, Inc., San Diego, CA).

Acquisition was performed at 2 minutes per sample. Flow rate was set at 14 μL/min. Forward scatter (FSC), side scatter (SSC) and fluorescence data were obtained with the settings in the logarithmic scale. MP gate was set as described in the S1 Appendix. In brief, the upper threshold for FSC and SSC to ≤1 μm was set with the Megamix-Plus FSC beads (BioCytex, Marseille, France, Fig A in S1 Appendix). Megamix-Plus FSC beads for cytometer settings in microparticle analysis are a mix of beads of the following bead-equivalent diameters: 0.1 μm, 0.3 μm, 0.5 μm and 0.9 μm. According to beads signal, the lower detection limit was placed as a threshold above the electronic background noise of the flow cytometer for FSC and approximately at the second logarithm for SSC (Figs A and C in S1 Appendix).

Data were analyzed with the BD CSampler software (version 1.0.264.21, Accuri^®^ Cytometers, Inc.). The cMP concentration (number of cMPs per μL of plasma) was determined according to Nieuwland’s formula[15], based on sample’s volume, flow cytometer’s flow rate and the number of fluorescence-positive events (N), as follows: cMPs/μL = N x (Vf/Va) x [Vt/(FRx 2)] x (1/Vi) [where Vf(μL) = final volume of washed cMP suspension, Va(μL) = volume of washed cMP suspension used for each labeling analysis, Vt(μL) = total volume of cMP suspension before fluorescence-activated cell sorting analysis, FR(μL/min) = flow rate of the cytometer at low mode (the average volume of microparticle suspension analyzed in one minute), 2 are the minutes of acquisition, 1 is the μL unit of volume, and Vi(μL) = original volume of plasma used for microparticle isolation].

### Plasma tissue factor activity measurement

Actichrome^®^ TF (ref 846, Sekisui Diagnostics, Stambford, CT) was used to measure TF-PCA in plasma samples as per manufacturer’s instructions.

### Statistical analyses

Sample size was determined with the ENE 3.0 statistical program (GlaxoSmithKline, Brentford, UK). We estimated that to have the power to detect mean differences in the number of CD61^+^/AV^+^ cMPs between STEMI and NSTEMI patients of 90 units with a conservative SD of 180, 85 subjects would be needed to complete the study (α risk = 0.05, power = 0.9). Thus, to obtain greater statistical power, the sample size was more than doubled. The number of CD61^+^/AV^+^ cMPs was used to determine the sample size but all cMPs were considered primary outcomes.

Statistical analyses were performed using the SPSS Statistical Analysis System (version 23.0, IBM Corp. Armonk, NY). Descriptive statistics [mean ± SD or n (%)] were used to describe the characteristics of the patients and the outcome variables. Normality of variables was assessed with the Shapiro-Wilk test. All variables with a skewed distribution were transformed to their natural logarithms for analyses. One-way ANOVA was used to assess the differences in cMPs and TF-PCA according to the type and severity of AMI. Linear regression was also applied to analyze the effects of the clinical characteristics during the acute event in the differences in cMPs and TF-PCA according to the type and severity of AMI. ROC curve analyses to identify the origin of cMPs associated to AMI severity were performed, and their corresponding C-statistics [areas under the curve (AUC) with their 95% confidence interval (CI)] were calculated. Correlation analyses between cMPs and TF-PCA were performed with Pearson’s correlation coefficient. A two-tail *P*-value of <0.05 was considered statistically significant.

## Results

### Patients’ characteristics

Table 1 shows the characteristics of the patients studied. The mean age was 75 years and 56.5% were hypertensive. A 42% of patients were hyperlipidemic, 22% diabetics, 10% current smokers, and 48.5% had history of cardiovascular disease (CVD, considered as a composite of previous angina, peripheral artery disease, AMI, stroke or heart failure). Finally, 15% of patients had suffered a previous malignant disease.

**Table 1:** Characteristics of the total population at inclusion in the study according to the type of myocardial infarction (n = 200).

	TOTAL (n = 200)	NSTEMI (n = 125)	STEMI (n = 75)	*P*
Males, n (%)	139 (69.5)	84 (67.2)	40 (53.3)	0.405
Age (years)	74.7 ± 3.8	75 ± 4	75 ± 4	0.764
BMI (Kg/m^2^)	25.9 ± 4.4	25.76 ± 3.50	26.44 ± 4.71	0.245
Systolic blood pressure (mm Hg)	138 ± 19	139 ± 20	137 ± 16	0.587
Diastolic blood pressure (mm Hg)	75 ± 10	75 ± 10	76 ± 9	0.396
Total cholesterol (mmol/L)	3.87 ± 0.82	3.93 ± 0.73	3.79 ± 0.94	0.255
HDL cholesterol (mmol/L)	1.40 ± 0.44	1.42 ± 0.43	1.35 ± 0.46	0.281
LDL cholesterol (mmol/L)	2.14 ± 0.63	2.17 ± 0.59	2.11 ± 0.69	0.535
Hypertension, n (%)	113 (56.5)	70 (56.0)	43 (57.3)	0.903
Dyslipidemia, n (%)	84 (42)	58 (46.4)	26 (34.7)	0.094
Diabetes, n (%)	44 (22)	28 (22.4)	16 (21.3)	0.837
Previous angina, n (%)	55 (27.5)	45 (36.0)	10 (13.3)	<0.0001
Previous peripheral artery disease, n (%)	14 (7)	10 (8.0)	0 (0.0)	0.012
Previous acute myocardial infarction, n (%)	58 (29)	44 (35.2)	14 (18.7)	0.011
Previous arrhythmia, n (%)	20 (10)	11 (8.8)	7 (9.3)	0.869
Previous stroke, n (%)	11 (5.5)	10 (8.0)	1 (1.3)	0.044
Previous heart failure, n (%)	9 (4.5)	8 (6.4)	1 (1.3)	0.092
Previous bleeding, n (%)	23 (11.5)	13 (10.4)	10 (13.3)	0.542
Previous malignant disease, n (%)	30 (15)	21 (16.8)	9 (12)	0.365
Current smokers, n (%)	19 (9.5)	12 (9.6)	7 (9.3)	0.936
Medication at inclusion, n (%)				
Acetylsalicylic acid	190 (95)	117 (93.6)	73 (97.3)	0.327
Antiplatelet agents	187 (93.5)	112 (89.6)	74 (98.7)	0.021
Statins	195 (97.5)	120 (96.)	75 (100.0)	0.116
Beta blocker	173 (86.5)	108 (86.4)	65 (86.7)	0.930
Calcium channel blocker	34 (17)	25 (20.0)	9 (12.0)	0.138
ACE inhibitor	64 (32)	34 (27.2)	30 (40.0)	0.066
ARB	49 (24.5)	33 (26.4)	16 (21.3)	0.402
Nitrates	21 (10.5)	18 (14.4)	3 (4.0)	0.019
Diuretics	42 (21)	28 (22.4)	14 (18.7)	0.512
Prednisolon	10 (5)	8 (6.4)	2 (2.7)	0.236
NSAIDs	4 (2)	3 (2.4)	1 (1.3)	0.597
Anticoagulants	25 (12.5)	15 (12)	10 (13.3)	0.799

Results are expressed as mean ± sd or n (%) when indicated. *P* from the comparison between STEMI and NSTEMI patients (*t*-test for unpaired samples for quantitative variables and Chi-squared test for qualitative variables). BMI indicates body mass index; HDL, high density lipoprotein; LDL, low density lipoprotein; ACE, angiotensin converting enzyme; ARB, angiotensin II receptor blockers; and NSAIDs, nonsteroidal anti-inflammatory drugs.

No differences were observed in the number of cMPs from any cell origin or in the plasma TF-PCA according to the presence of hypertension, hyperlipidemia, diabetes, previous CVD or previous malignant disease. In addition, no statistical differences in the number of cMPs from any cell origin were observed between samples taken at 2 or 8 weeks after the AMI.

From the 200 patients studied, 75 presented with STEMI (and 125 with NSTEMI), 17 patients presented with aHF (10 STEMI and 7 STEMI patients), and 23 were allocated at NYHA functional classification class III (16 NSTEMI patients -2 of them with aHF-, and 7 STEMI patients, from whom 2 presented with aHF as well).

### Circulating microparticles and AMI severity

**Fig 1:**
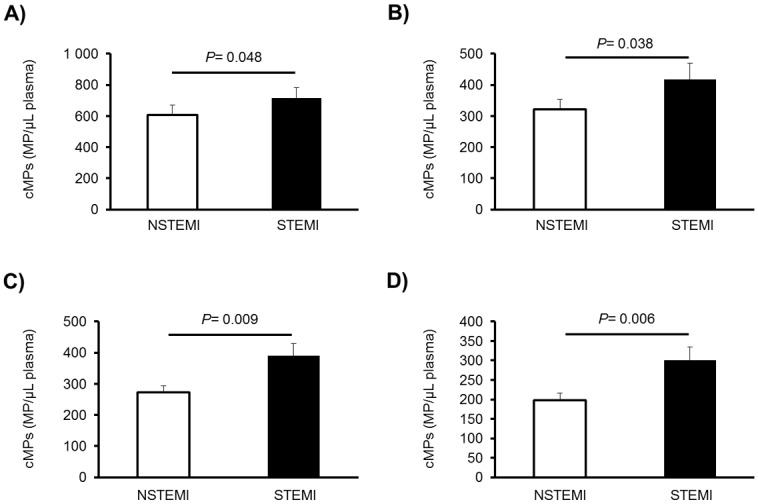
Differences in cMPs between STEMI and NSTEMI patients after 2–8 weeks of suffering an AMI. Results are expressed as mean + SEM. P values from the differences between STEMI (n = 75) and NSTEMI patients (n = 125, one-way ANOVA). cMPS denote circulating microparticles; STEMI, ST-elevation myocardial infarction; PFP, platelet-free plasma; and AV, Annexin V. A) CD61^+^/AV^+^ cMPs, where CD61 is a biomarker of platelets; B) CD31^+^/AV^+^ cMPs, where CD31 is PECAM (platelet/endothelial cell adhesion molecule 1); C) CD42b^+^/AV^+^ cMPs, where CD42b is von Willebrand factor receptor; and D) CD31^+^/CD42b^+^/AV^+^ cMPs, positive for both CD31 and CD42b.

Clinical characteristics during the acute event according to the type of myocardial infarction can be observed in Table C in S1 Appendix. STEMI patients (n = 75) showed significantly higher levels of platelet-derived cMPs (~18% CD61^+^/AV^+^, ~30% CD31^+^/AV^+^, ~43% CD42b^+^/AV^+^ and ~52% CD31^+^/CD42b^+^/AV^+^, *P* = 0.048, 0.038, 0.009 and 0.006, respectively), compared to NSTEMI patients (Fig 1), despite being under more intensive dual antiplatelet therapy (Table C in S1 Appendix).

The ROC-curve analysis indicated that CD31^+^/CD42b^+^/AV^+^ cMPs [*P* = 0.004; AUC = 0.622 (95% CI, 0.542, 0.703)] were the cMPs mostly related to STEMI among the AMI patients. These cMPs are shed by platelets and may indicate that platelet activation is more involved in STEMI than in NSTEMI development. As expected,~360% and ~100% higher levels of peak TnT and NT-proBNP were recorded in STEMI compared to NSTEMI patients [(n = 125) *P*<0.0001, both].

**Fig 2:**
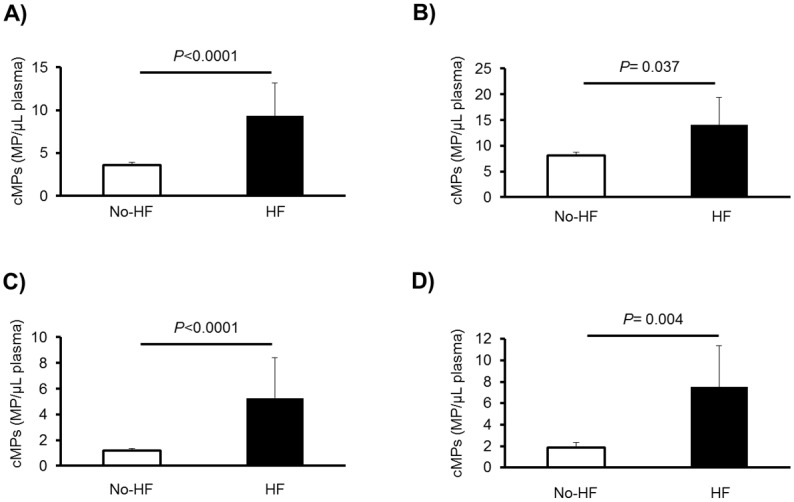
Differences in cMPs between patients who suffered a HF during the AMI and patients who did not. Results are expressed as mean + SEM. *P* values indicate the differences from one-way ANOVA analyses between patients who suffered a HF during the AMI (HF, n = 17) and patients who did not (No-HF, n = 183). cMPS denote circulating microparticles; HF indicates heart failure; AMI, acute myocardial infarction; PFP, platelet-free plasma; and AV,Annexin V. A) CD61^+^/CD142^+^/AV^+^ cMPs, where CD61 is a biomarker of platelets and CD142, tissue factor; B) CD14^+^/AV^+^ cMPs, where CD14 is a biomarker of monocytes; C) CD14^+^/CD11b^+^/AV^+^ cMPs, where CD11b is macrophage-1 antigen (Mac-1); and D) CD14^+^/CD142^+^/AV^+^ cMPs, monocyte-derived cMPs carrying tissue factor.

Patients who suffered acute heart failure (aHF) during AMI (n = 17) presented ~160% and ~300% increased levels of platelet (CD61^+^)- and monocyte (CD14^+^)-derived cMPs carrying TF (CD142^+^) (p<0.0001 and 0.004 for CD61^+^/CD142^+^/AV^+^ and CD14^+^/CD142^+^/AV^+^, respectively) (Fig 2), as well as ~87% and ~103% increased levels of peak TnT (*P* = 0.038) and NT-proBNP (*P* = 0.005) compared to those without aHF during AMI. However, no specific cMPs were significantly associated with presentation of aHF, as the highest c-statistic, found with cMPs carrying CD14^+^/CD142^+^/AV^+^, was not statistically significant [*P* = 0.069 AUC = 0.634 (95% CI, 0.491, 0.776)].

**Fig 3:**
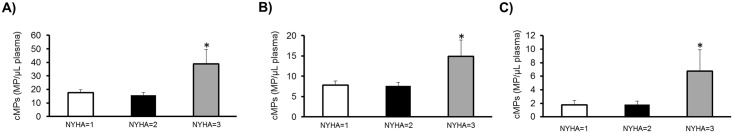
Differences in cMPs according to the NHYA classification. Results are expressed as mean + SEM. A) CD142^+^/AV^+^ cMPs, where CD142 is tissue factor; B) CD14^+^/AV^+^ cMPs, where CD14 is a biomarker of monocytes; and C) CD14^+^/CD142^+^/AV^+^ cMPs, monocyte-derived cMPs carrying tissue factor. *Significantly different from class I and II (*P* = 0.001, 0.015 and 0.014 for CD142^+^/AV^+^, CD14^+^/AV^+^ and CD14^+^/CD142^+^/AV^+^ cMPs respectively, one-way ANOVA with the Bonferroni posthoc test). According to the NHYA classification, 107 patients were allocated at class I, 70 at class II and 23 at class III. cMPs denote circulating microparticles; PFP, platelet-free plasma; and AV, Annexin V.

Patients in NYHA functional classification class III (n = 23) at inclusion also showed ~145%, ~96% and ~274% higher levels of CD142^+^/AV^+^, CD14^+^/AV^+^ and CD14^+^/CD142^+^/AV^+^ cMPs compared to class I and II patients [*P* = 0.001, 0.015 and 0.014, respectively, one-way ANOVA], as depicted in Fig 3. Monocyte cMPS carrying TF (CD14^+^/CD142^+^/AV^+^) [*P*<0.0001 AUC = 0.746 (95% CI, 0.632, 0.860)] are the most highly associated to NHYA class III.

No differences in cMPs of any cell origin were observed according to the other clinical characteristics or medication during the acute event or at inclusion. Although some clinical characteristics in the acute event differ between NSTEMI and STEMI, introducing these covariates in the regression model did not significantly alter the results. Finally, no correlations were found between any cMP and TnT or NT-proBNP concentrations.

### TF-PCA and AMI severity

STEMI patients showed 20% higher plasma TF-PCA compared to NSTEMI patients (*P* = 0.028, one-way ANOVA), but no differences were observed between patients who suffered aHF and patients who did not, or according to the NHYA classification.

In order to assess the procoagulant activity of the cMPs and especially TF-carrying cMPs, the correlation between circulating microparticles and TF-PCA was analyzed. As shown in Table 2, cMPs derived from platelets, monocytes and those carrying TF were positively correlated to plasma TF-PCA (r≥0.166, *P*≤0.027, all), indicating that these cMPs elicit a procoagulant-inducing activity.

**Table 2:** Correlations between cMPs and plasma TF-PCA in the study subjects (n = 200).

Correlations with TF-PCA		
	r*	P*
***Platelet-derived cMPs***		
**CD61**^**+**^**/AV**^**+**^	0.423	<0.0001
**CD31**^**+**^**/AV**^**+**^	0.393	<0.0001
**CD42b**^**+**^**/AV**^**+**^	0.440	<0.0001
**CD31**^**+**^**/CD42b**^**+**^**/AV**^**+**^	0.413	<0.0001
***Monocyte-derived cMPs***		
**CD14**^**+**^**/AV**^**+**^	0.278	<0.0001
***Tissue factor-carrying cMPs***		
**CD142**^**+**^**/AV**^**+**^	0.184	0.009
**CD142**^**+**^**/CD61**^**+**^**/AV**^**+**^	0.166	0.027
**CD142**^**+**^**/CD14**^**+**^**/AV**^**+**^	0.171	0.016

*r and *P from the Pearson’s correlation analyses.

TF-PCA denotes tissue factor-procoagulant activity; cMPs, circulating microparticles; AV, annexin V; and CD142, tissue factor. The other markers used for MP characterization are defined in Table A in S1 Appendix.

## Discussion

The main finding of our study was that cMPs from platelets, monocytes and cMPs bearing TF were increased in patients with STEMI versus NSTEMI, as well as in those developing HF during the AMI. Therefore cMPs are raised in patients with severe AMI presentation, with no correlation to peak TnT or NT-proBNP levels.

Increased numbers of platelet-shed cMPs were found in STEMI patients compared to NSTEMI[16]; and CD31^+^ and CD31^+^/CD42b^+^ cMPs were found unrelated to infarct size but correlated to the size of myocardium at risk[17]. However, in that particular study infarct size was measured by cardiovascular magnetic resonance imaging and not estimated by TnT (or NT-proBNP) levels. Our results indicate that cMPs from both platelets and monocytes provide information of the ongoing cell activation processes during AMI that are different from the TnT and NT-proBNP pathways.

During the acute phase of AMI, it is well known that platelets are activated[18] and, in their turn, activate monocytes and TF expression[19]. In addition, platelet cMPs exhibit significant procoagulant activity[20] due to phospholipid/charge structural membrane remodeling, and the presence of TF increases their pro-coagulant potential. Indeed, we observed a strong correlation between TF-PCA and platelet-derived cMPs. Platelet-derived cMPs account for around 84% of the total PS-exposing (AV^+^) cMPs, while TF-carrying cMPs accounts for approximately 2.5%. Phospholipids such as PS are critically important for the PCA of TF, and greatly accelerate TF-PCA[21]. Along this line, recent findings have pointed out the pivotal role of PS exposure at the MP surface in TF-PCA in NSTEMI patients[22], although the responsible mechanism is still under debate[23]. Nevertheless, we also found significant correlations between monocyte-derived and TF-carrying cMPs and plasma TF-PCA, indicating that these cMPs probably constitute the second largest pool of thrombogenic cMPs after platelet-derived MPs, as previously observed[24, 25].

Overall, increased platelet-, monocyte- and TF^+^-cMPs may reflect the interactions between platelets, monocytes and endothelial cells[26], that play an important role in the pathogenesis of thrombotic events leading to myocardial ischemia and may potentially determine AMI severity and prognosis at the long term. This is supported by the reports showing that AMI patients, independently of the severity of the event, present with increased platelet-[27, 28] and monocyte-derived cMPs[9], despite being under antiplatelet therapy[29]. Considering that TF-carrying cMPs constitute the principal reservoir of plasma TF activity and are associated with higher AMI severity, as shown in our study, as well as with cardiovascular mortality[8], these MPs may reflect long-term ongoing processes and a potential target for improving secondary prevention in AMI patients. Indeed, in STEMI patients we have observed that TF-carrying monocyte-derived cMPs are related to long-term prognosis of mortality for cardiovascular causes[29].

No differences were observed in cMPs derived from erythrocytes, from additional innate/adaptive immunity cells or from SMC, indicating that MPs from different cell origins exert different pathophysiological roles in the evolution of atherothrombosis.

STEMI patients showed 20% higher plasma procoagulant activity compared to NSTEMI patients, suggesting that STEMI patients are at higher risk of coronary thrombosis than NSTEMI patients, which more often suffer from atherosclerotic diseases. Unfortunately, we do not have enough data on the cause of AMI in those patients to confirm this hypothesis.

One of the strengths of our study resides in its sample size. Among AMI patients, reported incidences of STEMI (24% to 40%) and aHF during AMI (~17%)[30], are close to the proportions observed in our study, although the number of patients with aHF during AMI and at NYHA III classification is relatively low. Therefore, our study population is assumed to be representative for AMI patients, especially of elderly patients, a population subset in continuous expansion. Given the nature of AMI and because of infrastructural limitations, unfortunately we have no data on MP shedding in the acute phase. Nevertheless, the fact that 2–8 weeks after the AMI, platelet-, monocyte-derived and tissue factor-carrying circulating microparticles remain elevated reinforces our hypothesis that may relate to long-term ongoing process and may be of importance for long-term prognosis. Thus, our study is observational and hypothesis generating and further studies are needed for unveiling the underlying mechanisms by which MP are involved in post-AMI process.

Concluding, cMPs from platelets and monocytes and cMPs carrying TF discriminate AMI severity and the prothrombotic state, and may reflect platelet and monocyte activation during the acute phase of AMI. Therefore, TF-carrying, platelet and monocyte cMPs may reflect AMI severity and middle to long-term thrombotic burden.

## Supporting information

S1 Appendix **Table A. Cell surface molecules for circulating microparticle identification and characterization**. mAb indicates monoclonal antibody; PS, phosphatidylserine; LPS, lipopolysaccharide; APC, allophycocyanin; FITC, fluorescein isothiocyanate and PE, phycoerythrin. **Table B. Antibody panel for circulating microparticle identification and characterization**. Circulating microparticles were characterized with biomarkers of cell origin and biomarkers of cell activation as shown in S1 Table. mAb indicates monoclonal antibody; FITC, fluorescein isothiocyanate and PE, phycoerythrin. **Table C. Clinical characteristics in the acute phase of the event from the patients studied according to the type of myocardial infarction (n = 200)**. *P* from the comparison between STEMI and STEMI patients (*t*-test for unpaired samples for quantitative variables and Chi-squared test for qualitative variables). STEMI denotes ST-elevation myocardial infarction; PCI, percutaneous coronary intervention with stent implantation; HF, heart failure; VT/VFIB, ventricular arrhythmias/ventricular fibrillation; ICD, Implantable Cardioverter-Defibrillator; and LAD, left anterior descending. **Fig A. Gate limits for microparticle analysis with the Megamix-Plus FSC beads for cytometer settings in microparticle analysis**. A) According to Megamix-Plus FSC beads signal, the lower limit of quantification is >0.1μm, as beads of 0.1μm were negative for FITC signal (FL1 channel, positive fluorescence signal threshold established at the third logarithm of fluorescence intensity). B) Gate limits in the FSC/SSC plot for microparticle quantification were set according to gated beads signal. **Fig B. Upper gate limits for microparticle analysis**. A) Platelet region (P2) according to CD61 positive (^+^) events from platelet rich plasma (PRP) acquired 2 minutes with the settings for microparticle (MP) analyses, and MP region (P1) according to Megamix-Plus FSC beads signal (Fig A(b) in S1 Appendix) excluding platelet gate (P2). Certain degree of overlapping between platelet MPs and platelets is expected because small platelets size is similar to that of the largest platelet MPs (4). B) CD61 (platelet biomarker) staining from total events (P1 -MP gate- and P2 –platelet gate-). C) Annexin V (AV) staining of total events (P1 and P2). D) CD61 staining of AV negative events (platelets). **Fig C. Circulating microparticles identification and characterization with the AccuriC6 flow cytometer**. Representative plots for MP identification and characterization. A) P1 was set according to cMPs size and granularity (defined as <1μm, and Figs A and B in S1 Appendix). B) Annexin V-APC^+^ cMPs (M1) were selected from P1. C) AV^+^ cMPs binding PE^+^ (M2) or D) FITC^+^ (M3) labelled antibodies were selected from P1 and quantified. Double staining with FITC- and PE- labelled antibodies from M1 (Annexin V^+^ cMPs) was also quantified. APC denotes allophycocyanin; FITC indicates fluorescein isothiocyanate; and PE, phycoerythrin.
(DOCX)

S2 Appendix OMEMI trial design. Kristian Laake, Peder Myhre, Linn M Nordby, Ingebjørg Seljeflot, Michael Abdelnoor, Pål Smith, Arnljot Tveit, Harald Arnesen and Svein Solheim. Effects of omega 3 supplementation in elderly patients with acute myocardial infarction: design of a prospective randomized placebo controlled study. *BMC Geriatrics* 2014, 14:74.
(PDF)

S3 Appendix Dataset of circulating microparticles quantification. (PDF)
