# Supplementary material for: Platelet-, monocyte-derived and tissue factor-carrying circulating microparticles are related to acute myocardial infarction severity
Source: PLoS One. 2017 Feb 16;12(2):e0172558. doi: 10.1371/journal.pone.0172558 (PMC5313202; doi:10.1371/journal.pone.0172558)
Supplement: S1 Appendix — Table A. Cell surface molecules for circulating microparticle identification and characterization. mAb indicates monoclonal antibody; PS, phosphatidylserine; LPS, lipopolysaccharide; APC, allophycocyanin; FITC, fluorescein isothiocyanate and PE, phycoerythrin. Table B. Antibody panel for circulating microparticle identification and characterization. Circulating microparticles were characterized with biomarkers of cell origin and biomarkers of cell activation as shown in S1 Table. mAb indicates monoclonal antibody; FITC, fluorescein isothiocyanate and PE, phycoerythrin. Table C. Clinical characteristics in the acute phase of the event from the patients studied according to the type of myocardial infarction (n = 200). P from the comparison between STEMI and STEMI patients (t-test for unpaired samples for quantitative variables and Chi-squared test for qualitative variables). STEMI denotes ST-elevation myocardial infarction; PCI, percutaneous coronary intervention with stent implantation; HF, heart failure; VT/VFIB, ventricular arrhythmias/ventricular fibrillation; ICD, Implantable Cardioverter-Defibrillator; and LAD, left anterior descending. Fig A. Gate limits for microparticle analysis with the Megamix-Plus FSC beads for cytometer settings in microparticle analysis. A) According to Megamix-Plus FSC beads signal, the lower limit of quantification is >0.1μm, as beads of 0.1μm were negative for FITC signal (FL1 channel, positive fluorescence signal threshold established at the third logarithm of fluorescence intensity). B) Gate limits in the FSC/SSC plot for microparticle quantification were set according to gated beads signal. Fig B. Upper gate limits for microparticle analysis. A) Platelet region (P2) according to CD61 positive (+) events from platelet rich plasma (PRP) acquired 2 minutes with the settings for microparticle (MP) analyses, and MP region (P1) according to Megamix-Plus FSC beads signal (Fig A(b) in S1 Appendix) excluding platelet gate (P2). Certain degr [file pone.0172558.s001.docx]

**S1 Appendix**

**Platelet-, monocyte-derived and tissue factor-carrying circulating microparticles are related to AMI severity**

Gemma Chiva-Blanch, Kristian Laake, Peder Myhre, Vibeke Bratseth, Harald Arnesen, Svein Solheim, Lina Badimon and Ingebjørg Seljeflot.

**Table A. Cell surface molecules for circulating microparticle identification and characterization.**

| **mAb** | **Alternative name** | **Expression** | **Conjugation** | **Clone** | **Company** |
| --- | --- | --- | --- | --- | --- |
| Annexin V | PS-binding protein | Widely expressed | APC | - | BD Pharmingen |
| CD142 | Tissue Factor | Widely expressed | FITC | VD8 | Sekisui diagnostics |
| CD61 | β_3_-integrin | Platelets | PE | VI-PL2 | BD Pharmingen |
| CD31 | Platelet endothelial cell adhesion molecule | Activated Cells | FITC | AC128 | Miltenyi Biotec |
| CD62P | P-Selectin | Activated Platelets | FITC | AK-4 | BD Pharmingen |
| CD42b | Receptor for von Willebrand factor | Activated Platelets | PE | REA185 | Miltenyi Biotec |
| CD146 | Melanoma Cell Adhesion Molecule | Endothelial Cells | FITC | P1H12 | BD Pharmingen |
| CD62E | E-Selectin | Endothelial Cells | PE | 68-5H11 | BD Pharmingen |
| CD309 | Vascular endothelial growth factor receptor-2 | Endothelial Cells | FITC | ES8-20E6 | Miltenyi Biotec |
| CD45 | Leukocyte Common Antigen | Leukocytes | PE | Immu19.2 | Beckman Coulter |
| CD11b | Lymphocyte function-associated antigen 1 | Leukocytes | FITC | M1/70.15.11.5 | Miltenyi Biotec |
| CD62L | L-Selectin | Leukocytes | PE | LT-TD180 | Immunotools |
| CD14 | LPS-receptor | Macrophages, monocytes | PE | M5E2 | BD Pharmingen |
| CD3 | T-cell co-receptor | T-Lymphocytes | FITC | HIT3a | BD Pharmingen |
| CD235ab | Glycoporin A and B | Erythrocytes | FITC | HIR2 | Immunotools |
| SMA-α | Smooth Muscle Actin α | Smooth muscle cells | PE | 1A4 | R&D Systems |

mAb indicates monoclonal antibody; PS, phosphatidylserine; LPS, lipopolysaccharide; APC, allophycocyanin; FITC, fluorescein isothiocyanate and PE, phycoerythrin.

**Table B. Antibody panel for circulating microparticle identification and characterization.**

| **FITC-mAb** | **PE-mAb** | **Cell origin** |
| --- | --- | --- |
| CD142 | CD61 | Platelets |
| CD62P | CD62L | Platelets |
| CD146 | CD62E | Endothelial cells |
| CD31 | CD42b | Endothelial cells |
| CD235ab | - | Erythrocytes |
| CD3 | CD45 | Lymphocytes |
| CD11b | CD14 | Monocytes |
| CD142* | CD14 | Monocytes |
| CD142* | SMA-α | Smooth muscle cells |

Circulating microparticles were characterized with biomarkers of cell origin and biomarkers of cell activation as shown in S1 Table. mAb indicates monoclonal antibody; FITC, fluorescein isothiocyanate and PE, phycoerythrin.

**MP gating in the flow cytometer**

The upper threshold for FSC and SSC to ≤1 µm was set with the Megamix-Plus FSC beads (BioCytex, Marseille, France, S1 Fig). Megamix-Plus FSC beads for cytometer settings in microparticle analysis are a mix of beads of the following bead-equivalent diameters: 0.1 µm, 0.3 µm, 0.5 µm and 0.9 µm. According to beads signal, the lower detection limit was placed as a threshold above the electronic background noise of the flow cytometer for FSC and approximately at the second logarithm for SSC.

**Fig A. Gate limits for microparticle analysis with the Megamix-Plus FSC beads for cytometer settings in microparticle analysis.**


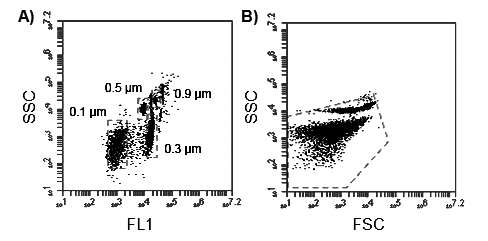


A) According to Megamix-Plus FSC beads signal, the lower limit of quantification is >0.1µm, as beads of 0.1µm were negative for FITC signal (FL1 channel, positive fluorescence signal threshold established at the third logarithm of fluorescence intensity). B) Gate limits in the FSC/SSC plot for microparticle quantification were set according to gated beads signal.

As shown in S2 Fig, in order to confirm that the gate for MP detection did not overlap with platelets gate, 10 µL of platelet rich plasma (PRP) obtained by centrifuging citrated peripheral blood 160xg 20 min at RT, was diluted in 100µL of Tyrode’s buffer (supplemented with glucose 5mmol/L, HEPES 20mmol/L and CaCl_2_ 2mmol/L, pH 7.3). Thereafter, 25 µL of PRP were incubated with 10 µL of CD61-PE and AV-APC in a final volume of 100 µL Tyrode’s buffer 20 min at RT. Binding reaction was stopped with 400 µL of ABB, prior to flow cytometer analyses with the settings for MP analysis.

**Fig B. Upper gate limits for microparticle analysis.**


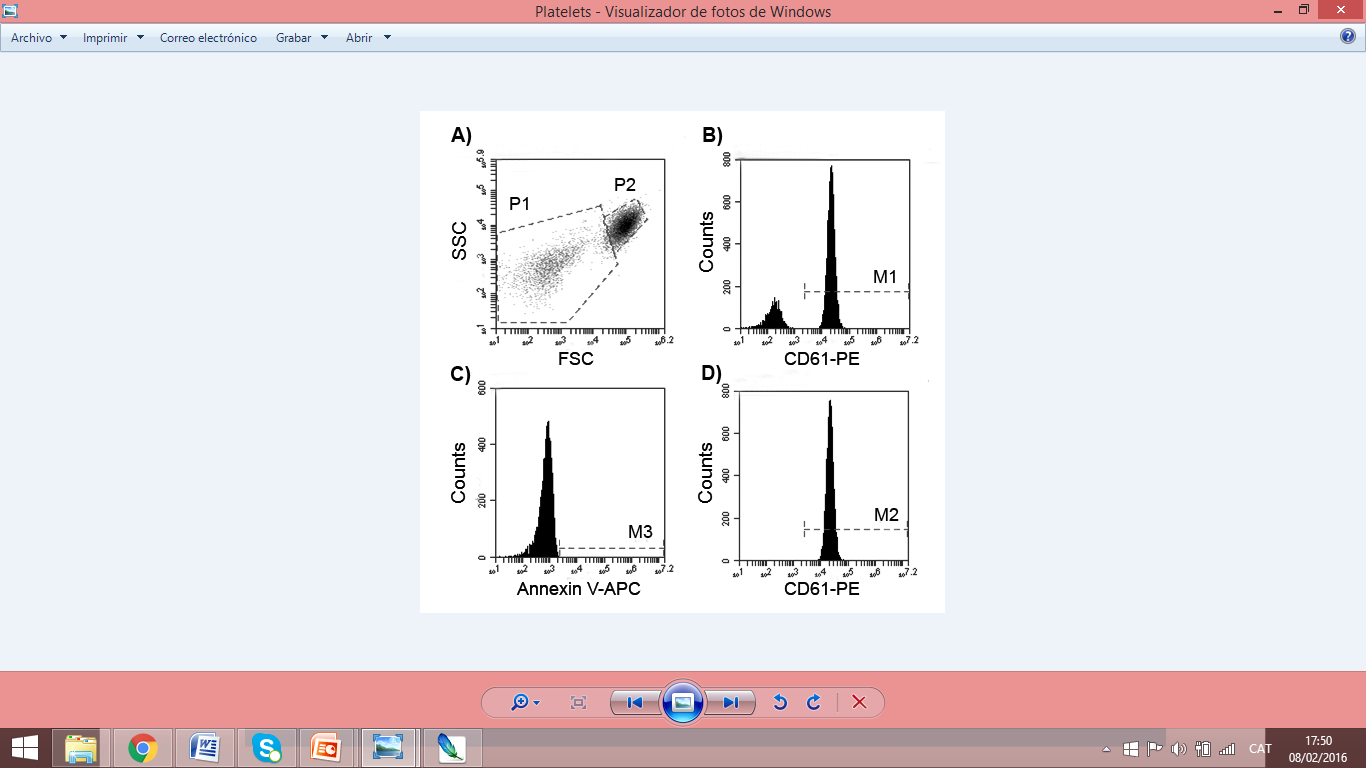


A) Platelet region (P2) according to CD61 positive (^+^) events from platelet rich plasma (PRP) acquired 2 minutes with the settings for microparticle (MP) analyses, and MP region (P1) according to Megamix-Plus FSC beads signal (S1 Fig B) excluding platelet gate (P2). Certain degree of overlapping between platelet MPs and platelets is expected because small platelets size is similar to that of the largest platelet MPs (4). B) CD61 (platelet biomarker) staining from total events (P1 -MP gate- and P2 –platelet gate-). C) Annexin V (AV) staining of total events (P1 and P2). D) CD61 staining of AV negative events (platelets).

cMPs within the established gate limits (>0.1 to ≤1 µm) were identified and quantified based on their binding to AV (that has a high specificity for PS) and reactivity to cell-specific mAb (S3 Fig). To identify positive marked events, thresholds of fluorescence were also set based on samples incubated with the same final concentration of isotype-matched control antibodies after titration experiments. AV binding level was corrected for autofluorescence using fluorescence signals obtained with microparticles in a calcium-free buffer (PBS). To reduce background noise, buffers were prepared on the same day and filtered through 0.2 µm pore size filters under vacuum.

cMPs derived from leukocytes other than lymphocytes (CD3^+^) and monocytes (CD14^+^) were inferred by subtracting lymphocyte- and monocyte-derived cMPs from total leukocyte (CD45^+^)-derived cMPs instead of labelling with specific mAb, and therefore defined as CD45^+^/CD3^-^/CD14^-^.

**Fig C. Circulating microparticles identification and characterization with the AccuriC6 flow cytometer.**


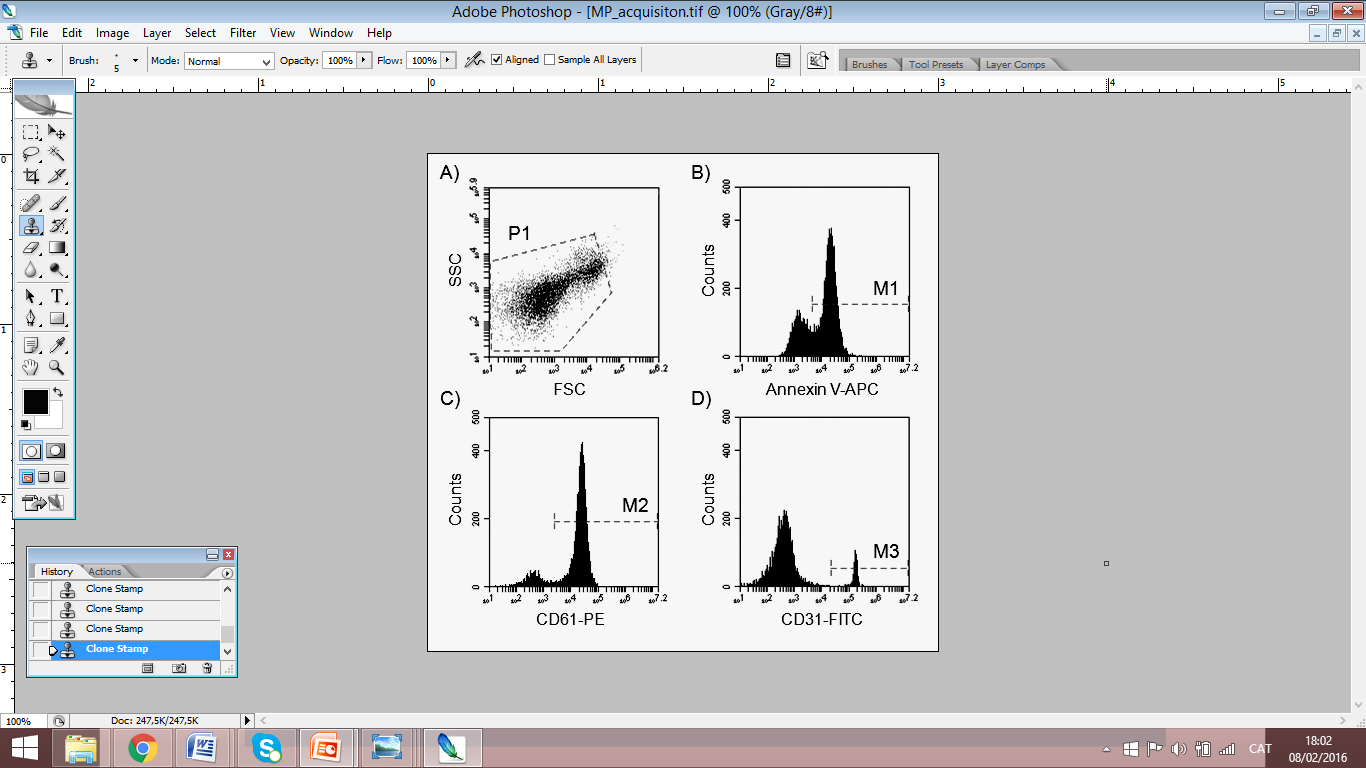


Representative plots for MP identification and characterization. A) P1 was set according to cMPs size and granularity (defined as <1µm, and S1 and S2 Figs). B) Annexin V-APC^+^ cMPs (M1) were selected from P1. C) AV^+^ cMPs binding PE^+^ (M2) or D) FITC^+^ (M3) labelled antibodies were selected from P1 and quantified. Double staining with FITC- and PE- labelled antibodies from M1 (Annexin V^+^ cMPs) was also quantified. APC denotes allophycocyanin; FITC indicates fluorescein isothiocyanate; and PE, phycoerythrin.

**Table C. Clinical characteristics in the acute phase of the event from the patients studied according to the type of myocardial infarction (n=200).**

|  |  | **NSTEMI (n=125)** | **STEMI (n= 75)** | ***P*** |
| --- | --- | --- | --- | --- |
| Thrombolytics |  | 0 (0) | 6 (8) | 0.006 |
| Dual antiplatelet therapy |  | 109 (87.2) | 72 (96) | 0.054 |
| Acetylsalicylic acid |  | 117 (93.6) | 73 (97.3) | 0.327 |
| Antiplatelet agents |  |  |  |  |
| Clopidogrel |  | 36 (28.8) | 46 (61.3) | <0.0001 |
| Prasugrel |  | 4 (3.2) | 21 (28) | <0.0001 |
| Tricagrelor |  | 72 (57.6) | 6 (8) | <0.0001 |
| PCI |  | 78 (62.4) | 68 (90) | <0.0001 |
| acute HF |  | 7 (5.6) | 10 (13.3) | 0.060 |
| Atrial fibrillation |  | 23 (18.4) | 13 (17.3) | 0.829 |
| paroxysmal |  | 17 (13.6) | 8 (10.7) | 0.626 |
| persistent |  | 6 (4.8) | 5 (6.7) | 0.441 |
| Episodes of VT/VFIB |  | 8 (6.4) | 21 (28) | <0.0001 |
| ICD |  | 2 (1.6) | 1 (1.3) | 0.875 |
| Pacemaker |  | 2 (1.6) | 3 (4) | 0.297 |
| Number of vessels >50% stenosis | |  |  |  |
| 1 |  | 47 (37.6) | 37 (49.3) | 0.055 |
| 2 |  | 32 (25.6) | 20 (26.7) | 0.380 |
| 3 |  | 30 (24) | 17 (22.7) | 0.814 |
| Infarct related artery |  |  |  |  |
| LAD artery |  | 47 (37.6) | 31 (41.3) | 0.001 |
| circumflex artery |  | 25 (20) | 10 (13.3) | 0.049 |
| right coronary artery |  | 25 (20) | 33 (44) | 0.009 |

*P* from the comparison between STEMI and NSTEMI patients (*t-*test for unpaired samples for quantitative variables and Chi-squared test for qualitative variables). STEMI denotes ST-elevation myocardial infarction; PCI, percutaneous coronary intervention with stent implantation; HF, heart failure; VT/VFIB, ventricular arrhythmias/ventricular fibrillation; ICD, Implantable Cardioverter-Defibrillator; and LAD, left anterior descending.
