# Supplementary material for: Platelet-, monocyte-derived and tissue factor-carrying circulating microparticles are related to acute myocardial infarction severity
Source: PLoS One. 2017 Feb 16;12(2):e0172558. doi: 10.1371/journal.pone.0172558 (PMC5313202; doi:10.1371/journal.pone.0172558)
Supplement: S2 Appendix — Kristian Laake, Peder Myhre, Linn M Nordby, Ingebjørg Seljeflot, Michael Abdelnoor, Pål Smith, Arnljot Tveit, Harald Arnesen and Svein Solheim. Effects of omega 3 supplementation in elderly patients with acute myocardial infarction: design of a prospective randomized placebo controlled study. BMC Geriatrics 2014, 14:74. (PDF) [file pone.0172558.s002.pdf]

Laake et al. BMC Geriatrics 2014, 14:74
http://www.biomedcentral.com/1471-2318/14/74

STUDY PROTOCOL Open Access

Effects of omega 3 supplementation in elderly patients with acute myocardial infarction: design of a prospective randomized placebo controlled study

Kristian Laake[1,3*], Peder Myhre[2], Linn M Nordby[2], Ingebjørg Seljeflot[1,3,4], Michael Abdelnoor[4,5], Pål Smith[2], Arnljot Tveit[6], Harald Arnesen[1,4] and Svein Solheim[1,3]

Abstract

Background: Both epidemiological and randomized clinical studies suggest that supplementation with very-long-chain marine polyunsaturated n-3 fatty acids (n-3 PUFA) have cardioprotective effects, however these results are not without controversy. Study population, sample-size, type of supplementation and type of endpoint have all varied widely accross different studies.
Therefore, the aims of the present study are to evaluate the effect of 2 years supplementation with capsules of very-long chain marine n-3 PUFA on top of standard therapy in elderly patients after acute myocardial infarction (AMI).
In addition, special characteristics of this population with regard to prediction of clinical outcome will be investigated. The hypothesis is that this supplementation on top of modern therapy will reduce the occurence of major cardiovascular events (MACE). We present the design of the OMEMI (OMega-3 fatty acids in Elderly patients with Myocardial Infarction) study.

Methods/Design: The OMEMI study is designed as a randomized, placebo-controlled double-blind multicenter trial. Included are patients ≥70-82 years of age who have sustained AMI. Patients of either gender are eligible. Sample size calculation based on existing literature has resulted in the need for 1400 patients followed for 2 years, based on the assumption that the n-3 PUFA supplementation will reduce MACE with 30%. The study medication is Pikasol® Axellus AS, Norway, 3 capsules (1.8 g eicosapentaenoic acid (EPA) + docohexaenoic acid (DHA)) per day, and matching placebo is corn oil. The Primary end-point is the composite of total mortality, first non-fatal recurring AMI, stroke and revascularization. Secondary end-point is the occurrence of new onset atrial fibrillation. Extensive biobanking will be performed, including adipose tissue biopsies. Compliance will be assessed by measurements of the fatty acid profile in serum, sampled at inclusion, after 12 months and at the end of study.

Discussion: The OMEMI study is scheduled to terminate when the last included patient has been followed for 2 years. To the best of our knowledge, the OMEMI study is the first to evaluate the effect of n-3 PUFAs on CVDs and mortality in a high risk elderly population having suffered an acute myocardial infarction.

Trial registration: ClinicalTrials.gov, NCT01841944

Keywords: Omega 3 fatty acids, Acute myocardial infarction, Randomized placebo controlled trial, Elderly

* Correspondence: kristian.laake@ous-hf.no
[1]Center for Clinical Heart Research, Oslo University Hospital, Ullevål, Oslo, Norway
[3]Department of Cardiology, Oslo University Hospital, Ullevål, Oslo, Norway
Full list of author information is available at the end of the article

© 2014 Laake et al.; licensee BioMed Central Ltd. This is an Open Access article distributed under the terms of the Creative Commons Attribution License (http://creativecommons.org/licenses/by/4.0), which permits unrestricted use, distribution, and reproduction in any medium, provided the original work is properly credited. The Creative Commons Public Domain Dedication waiver (http://creativecommons.org/publicdomain/zero/1.0/) applies to the data made available in this article, unless otherwise stated.

Background

Acute myocardial infarction (AMI) is a major cause of mortality and morbidity in westernized countries [1,2]. With a mean age of individuals suffering an AMI of about 72 years, a major proportion of the patients are above 70 years [1-3]. Although the management of AMI has improved, still a significant number of patients, especially elderly, are at increased risk for adverse events.

The possible benefit of marine omega-3 polyunsaturated fatty acids (n-3 PUFAs) in the prevention of atherosclerosis, the main underlying process behind coronary heart disease (CHD) and an AMI, was first proposed by Dyerberg & Bang after their studies on the Greenland Eskimos in the 1970s [4-6].

Later, a considerable amount of research has been performed on the subject, and n-3 PUFAs have been shown, in some studies, to reduce morbidity and mortality in patients with various cardiovascular disease (CVD) states [7-11], also in elderly individuals [12]. In the DART trial [13], Burr et al. showed that 2033 post-AMI patients randomised to receive or not receive advice on dietary components, those adviced to eat fatty fish had a 29% reduction in 2 year all-cause mortality compared with those not adviced. In the GISSI-4 study [7] 1 g n-3 PUFA/day reduced sudden cardiac death by 40% after 3 years in patients after an AMI [8], and in the DOIT study [14], although not statistically powered for clinical end-points, a 50% reduction in mortality with supplementation of 2.4 g PUFAs/day was achieved after 3 years in elderly patients at high risk for CVD. In a more recent study, higher plasma levels of n-3 PUFAs in 2692 healthy older adults were associated with lower total mortality, especially CHD deaths [15].

However, data is still inconsistent. Recent clinical trials and meta-analyses on n-3 PUFA and CVD, have suggested limited effect [16-19], but the studies differ widely in dosage of study medication, population size, participants disease states and time of follow-up. The lack of effect might also be attributed to the state of art drug treatment not available in the older trials [20]. All these factors may contribute to the confusion in the field. Both the SU.FOL.OM3 [16] and OMEGA trial [17] are considered statistically underpowered [21]. The even larger Alpha Omega trial [18] with 4837 participants clinically diagnosed with myocardial infarction up to 10 years before randomization, showing no effects of n-3 PUFAs on CVD, has also been critized for being underpowered, for using low doses omega-3 and for the formulation used which may nullify any benificial effect [22]. The lack of unanimous agreement is typically shown in the 2 recent meta-analysis by Rizos et al. and Delgado-Lista et al., reviewing n-3 PUFAs effect on CVD including many of the same studies, but coming to opposite conclusions [23,24].

Results from studies on mechanisms have shown that the cardioprotective effects of n-3 PUFA acts through a range of processes involving triglyceride lowering, anti-inflammatory, antiarrhythmic and antithrombotic effects in addition to a beneficial influence on blood pressure, heart rate and endothelial function [25,26].

Methods/Design

The OMEMI (OMega-3 fatty acids in Elderly patients with Myocardial Infarction) study is a prospective, randomized, placebo-controlled, double blinded multicenter trial.

Aim of study

The aim of the study is to investigate the effects of supplementation with 1.8 g/day of n-3 PUFAs on cardiovascular morbidity and mortality during a follow-up period of 2 years in an elderly population after having experienced an AMI. In addition, special attention to the characteristics of this elderly population in general and with regard to prediction for clinical outcome will be investigated. An extensive biobank will be established, including adipose tissue samples as well as serum for specific fatty acid analysis. Our hypothesis is that supplementation of n-3 PUFAs will reduce the risk for cardiovascular events.

Patients and centres

Patients will be recruited from three hospitals in the Oslo area, Oslo University Hospital (OUS Ullevål) (study center), Akershus University Hospital (AHUH) and Vestre Viken Bærum Hospital (VVBH). All patients with a diagnosis of AMI, being ≥70-82 years of age will be screened at the respective wards, and eligible patients will be asked for participation after oral and written information will be given. In accordance with the Declaration of Helsinki [27], written consent is obtained. Inclusion will be within 2–8 weeks after the acute myocardial infarction. The inclusion and exclusion criteria are given in Table 1. Permuted block randomization will be undertaken by use of consecutively numbered non-translucent envelopes containing allocation message to either of the randomized groups in a 1:1 ratio, arranged by the Unit of Epidemiology and Biostatistics, OUS, according to tables of random numbers, stratified by centers (OUS/AHUH and BH).

Drug regimens and treatment strategies

Patients will be treated according to general guidelines and continue standard medication during the study period. At inclusion the patients will be randomized to either n-3 PUFA or placebo. The patients randomized to the active treatment, will receive Pikasol®, 3 capsules (1.8 g EPA + DHA)/day, and those in the placebo group

Table 1 Inclusion and exclusion criteria in the OMEMI trial

Inclusion criteria	*Exclusion criteria*
- Diagnosis of acute AMI (type I,II,IV) according to current guidelines.	- Documented intolerance for Omega-3 fatty acids.
- Age 70–82, either gender.	- Comorbidity thought to be incompatible with compliance to study drugs.
- Not being part of another randomized trial.	- Comorbidity thought to reduce survival for the follow-up time of 2 years.
- Understand Norwegian and giving written consent to participate.	

will receive 3 identically designed capsules of corn oil. Corn oil has been used as placebo in many randomized studies on n-3 PUFAs in CVD. It is considered safe and without any known adverse effects or influence on atherothrombosis. Patients being on supplementation with n-3 fatty acids at inclusion will be permitted to continue with a daily dose of 1 child-spoon with cod-liver oil, but not with capsules containing n-3 fatty acids beyond the study drugs. The study drugs will be provided by Axellus AS (Oslo, Norway) and picked up by the participant in sealed containers from the respective hospital pharmacies at OUS and AHUH at each visit.

Study visits

The study design of the OMEMI trial is visualized in Figure 1A. The flow-chart of the OMEMI trial according to CONSORT 2010 (checklist in additional file 1) is visualized in Figure 1B. Baseline registration include medical history, use of medication and clinical examination. Participants will also be asked to fill out a food questionaire. ECG will be recorded at baseline, and at 12 and 24 months and a biobank will be established at all time points including fat biopsies at baseline and end of study.

Compliance will be asessed by measurements of the fatty acid profile in serum sampled at inclusion, 12 months and at end of study. The patients will be followed up for two years. Patients that for any reason have been off study medication for more than 4 consecutive weeks will be classified as drop-outs.

End points

The primary endpoint of the OMEMI study is the composite of total mortality, first event of non-fatal AMI, stroke and revascularization (MACE). Secondary endpoint is occurrence of new onset atrial fibrillation, registered by ECG at follow-ups, and from any hospital admissions during the study period. In addition, patients will be equipped with an electronic device manufactured by Zenicor Medical Systems AB (Stockholm, Sweden) at 1 year follow-up, which allow twice daily ECG recordings at home for 2 weeks of any occurence of atrial fibrillation.

Among patients who are not able to attend the last visit, the clinical end-points will be recorded by the investigator on request. Cause-specific mortality data will be obtained from death certificates provided from the Database of Statistics, Norway. Evaluation of end-points will be undertaken by an external End-point Committee. Internationally accepted diagnostic criteria will be used.

Substudies

Extensive biobanking will be undertaken according to accepted common sampling and processing protocols at all study visits. Samples will be frozen and create the basis for different biochemical substudies:

- Serum fatty acid profile for compliance and correlation analyses
- Special characteristics of this elderly population in general with regard to prediction for clinical outcome
- Antiplatelet therapy in the elderly
- Gender differences
- Effects of PCI/thrombolysis

Effects of n-3 PUFAs in the elderly on:

- Markers of Endothelial function; Inflammation; Coagulation and Fibrinolytic activity, measured as circulating proteins and mRNA in leukocytes
- Expression of relevant genes associated with atherothrombosis in adipose tissue
- Influence of genetic polymorphisms on atherothrombosis
- Heart rhythm (HRV) by use of a new available method (Zenicor Medical Systems AB)

Adverse events and monitoring

All adverse events, including gastroenteric complaints and possible interaction with other drugs that are used will be recorded. As a majority of the patients will be treated with antiplatelet drugs, special attention will be given to bleeding complications in this elderly population. Standardized bleeding definitions using the BARC criteria will be used [28]. Regular reports will be delivered to the Data Safety and Monitoring Board (DSMB). Serious adverse events (SAE) will be reported to the National Drug Authority. CIOMS (Council for International Organizations of Medical Sciences) form will be used for reporting SAE.

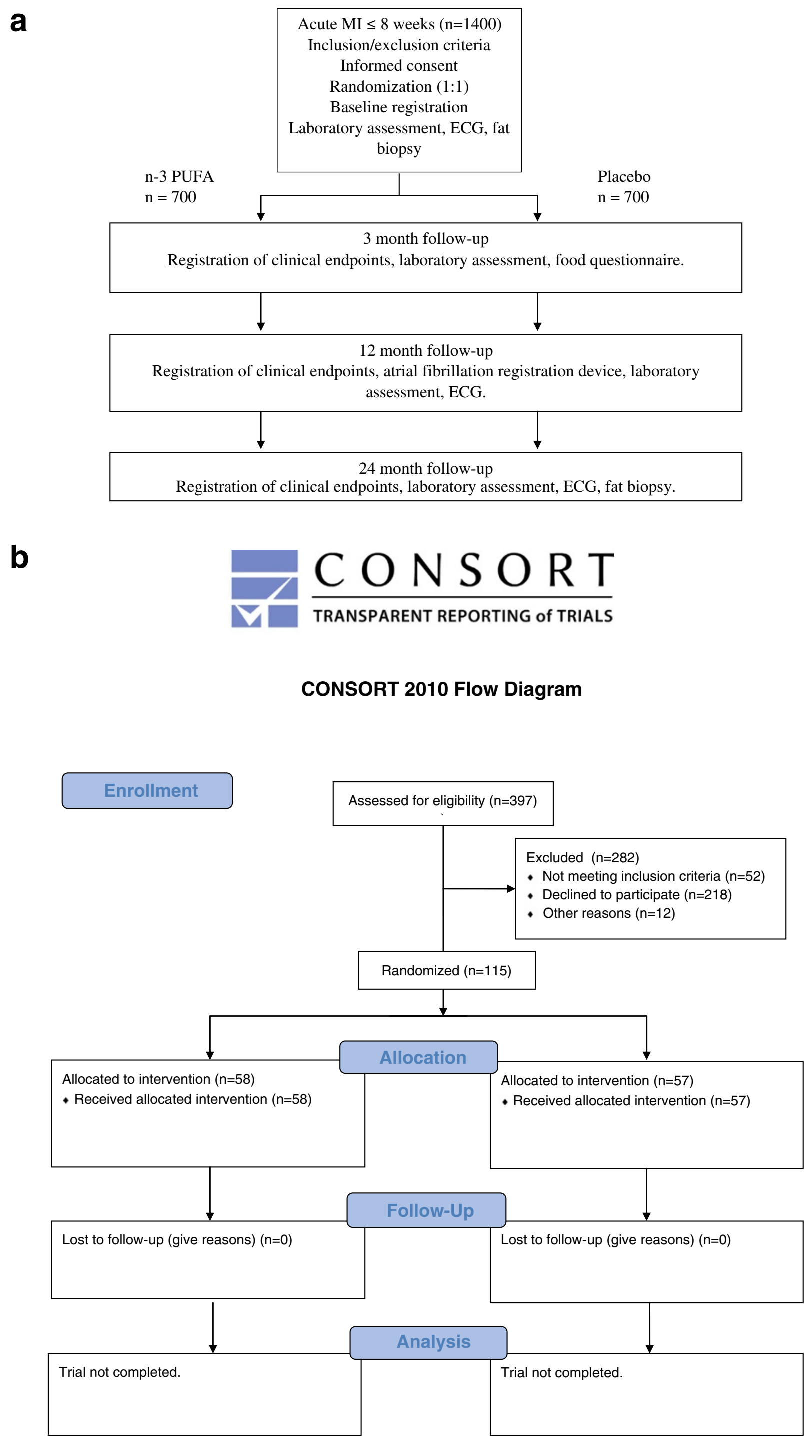

Figure 1 Design (a) and flow-chart according to CONSORT (b) in the OMEMI trial.

Statistical analyses

According to the results from the GISSI-4 and DOIT trials [7,14], we anticipate that the supplementation of n-3 PUFA on top of modern therapy will reduce the combined cardiovascular end-point of death, non-fatal AMI, stroke and revascularizations (MACE) by 30% in the active drug group. Based on data from registries [3,29] and with an alpha of 0.05 and a power of 80%, 611 patients would be needed in each study group. Allowing for an estimated drop-out rate of 10-15%, a total number of 1400 patients will be included. No interim analyses are planned, but the DSMB will have access to the total

Table 2 Baseline characteristics of the first 115 subjects at inclusion in the OMEMI trial; Data presented as percentages or median values (25, 75 percentiles)

Age (y)	75 (72, 78)
Gender (male/female) (%)	73.9/26.1
Current smoker (%)	11.3
BMI (kg/m^2)	25.5 (23.9, 28.1)
S-total cholesterol (mmol/L)	3.80 (3.20, 4.40)
S-LDL (mmol/L)	2.06 (1.69, 2.52)
S-HDL (mmol/L)	1.26 (1.03, 1.59)
S-triglycerides (mmol/L)	1.15 (0.88, 1.54)
Systolic blood pressure (mmHg)	135 (125, 150)
Pulse rate (bpm)	66 (60, 72)
STEMI (%)	33.9
Troponin-T (peak level) (ng/L)	735 (168, 2562)
Previous atrial fibrillation (%)	13.9
Previous myocardial infarction (n)	46 (40.0%)
Aspirin (%)	93.9
Clopidogrel (%)	40.8
Prasugrel (%)	12.1
Ticagrelor (%)	40.0
Anticoagulation (%)	16.5
Betablocker (%)	89.5
ACE-I/AT II blocker (%)	64.3
Calcium channel blocker (%)	21.7
Statin (%)	96.5
Diuretic (%)	27.8
Nitrates (%)	13.0
n-3 PUFA supplements (%)	47.3

ACE-I/AT II: angiotensin-converting enzyme inhibitors/angiotensin II receptor blockers; S-LDL: low density lipoprotein; S-HDL: high-density lipoprotein; STEMI: ST-segment elevation myocardial infarction.

number of primary end-points for potential prolongation of the study period or increase in the number of participants. Statistical analyses will primarily be performed according to the intention-to-treat principle.

Approval

The protocol has been approved by the Regional Committee for Medical Research Ethics (ref. number: 2012/1422), and the study has been registered at ClinicalTrials.gov, April 16th 2013, NCT01841944.

Administrative matters

The study has a Steering committee for monitoring the study progress and quality. All endpoints and SAE will be finally evaluated before closure of the study.

Discussion

The OMEMI study is scheduled to terminate when the last included patient has been followed for 2 years, probably in 2016/17. Selected baseline characteristics of the first 115 patients included are shown in Table 2. To the best of our knowledge, the OMEMI study is the first to evaluate the effect of n-3 PUFAs on CVDs and mortality in a high risk elderly population having suffered an acute myocardial infarction.

Additional file

Additional file 1: CONSORT 2010 checklist of information to include when reporting a randomised trial*.

Competing interests

The authors declare that they have no competing interests.

Authors' contributions

KL conducts the study and is responsible for analysis and interpretation of the data, drafted and revised the manuscript. PM and LMN conducts the study, will contribute to the interpretation of the results and revised the manuscript. MA made contributions to the statistics used, study protocol and design. IS and HA conceived of the study, contributed to the study protocol, obtained funding and discussed the manuscript. PS and AT contributed to the study design and protocol and assists in the data collection. SS coordinates the study and made substantial contribution to the study protocol, will contribute to data analysis and intepretation, and revised the manuscript. All the authors contributed to revision and approval of the final manuscript.

Acknowledgements

Financial support to the study is given by Stein Erik Hagens Foundation for Clinical Heart Research, Oslo, Norway. Applications for external grants will be adressed. Omega-3 capsules Pikasol® and matching placebo (corn oil) are provided by Axellus AS, Oslo, Norway.

Steering committee

Harald Arnesen MD PhD, Ingebjørg Seljeflot PhD, Svein Solheim MD PhD, Dan Atar MD PhD, Erik Berg Schmidt MD PhD, Pål Smith MD PhD, Arnljot Tveit MD PhD, Michael Abdelnoor PhD.

Author details

[1]Center for Clinical Heart Research, Oslo University Hospital, Ullevål, Oslo, Norway. [2]Department of Cardiology, Akershus University Hospital HF, Lørenskog, Norway. [3]Department of Cardiology, Oslo University Hospital, Ullevål, Oslo, Norway. [4]Faculty of Medicine, University of Oslo, Oslo, Norway. [5]Center of Clinical Research, Unit of Epidemiology and Biostatistics, Oslo University Hospital, Ullevål, Oslo, Norway. [6]Department of Medical Research, Vestre Viken Hospital Trust, Bærum Hospital, Rud, Norway.

Received: 11 February 2014 Accepted: 6 June 2014
Published: 13 June 2014

References

1. Mensah GA, Ryan US, Hooper WC, Engelgau MM, Callow AD, Kapuku GK, Mantovani A: **Vascular endothelium summary statement II: cardiovascular disease prevention and control.** *Vascul Pharmacol* 2007, **46**:318–320.
2. Hagen TP, Anthun KS, Reikvam A: **Acute myocardial infarctions in Norway 1.** *Tidsskr Nor Laegeforen* 2010, **130**:820–824.
3. Halvorsen S, Eritsland J, Abdelnoor M, Holst HC, Risoe C, Midtbo K, Bjornerheim R, Mangschau A: **Gender differences in management and outcome of acute myocardial infarctions treated in 2006–2007.** *Cardiology* 2009, **114**:83–88.
4. Dyerberg J, Bang HO, Stoffersen E, Moncada S, Vane JR: **Eicosapentaenoic acid and prevention of thrombosis and atherosclerosis?** *Lancet* 1978, **2**:117–119.

5. Dyerberg J, Bang HO: **Haemostatic function and platelet polyunsaturated fatty acids in Eskimos.** *Lancet* 1979, **2**:433–435.
6. Dyerberg J, Bang HO, Hjorne N: **Fatty acid composition of the plasma lipids in Greenland Eskimos.** *Am J Clin Nutr* 1975, **28**:958–966.
7. **Dietary supplementation with n-3 polyunsaturated fatty acids and vitamin E after myocardial infarction: results of the GISSI-Prevenzione trial. Gruppo Italiano per lo Studio della Sopravvivenza nell'Infarto miocardico.** *Lancet* 1999, **354**:447–455.
8. Marchioli R, Barzi F, Bomba E, Chieffo C, Di Gregorio D, Di Mascio R, Franzosi MG, Geraci E, Levantesi G, Maggioni AP, Mantini L, Marfisi RM, Mastrogiuseppe G, Mininni N, Nicolosi GL, Santini M, Schweiger C, Tavazzi L, Tognoni G, Tucci C, Valagussa F: **Early protection against sudden death by n-3 polyunsaturated fatty acids after myocardial infarction: time-course analysis of the results of the Gruppo Italiano per lo Studio della Sopravvivenza nell'Infarto Miocardico (GISSI)-Prevenzione.** *Circulation* 2002, **105**:1897–1903.
9. Tavazzi L, Maggioni AP, Marchioli R, Barlera S, Franzosi MG, Latini R, Lucci D, Nicolosi GL, Porcu M, Tognoni G: **Effect of n-3 polyunsaturated fatty acids in patients with chronic heart failure (the GISSI-HF trial): a randomised, double-blind, placebo-controlled trial.** *Lancet* 2008, **372**:1223–1230.
10. Nodari S, Triggiani M, Campia U, Manerba A, Milesi G, Cesana BM, Gheorghiade M, Dei CL: **Effects of n-3 polyunsaturated fatty acids on left ventricular function and functional capacity in patients with dilated cardiomyopathy.** *J Am Coll Cardiol* 2011, **57**:870–879.
11. De Caterina R: **n-3 fatty acids in cardiovascular disease.** *N Engl J Med* 2011, **364**:2439–2450.
12. Einvik G, Klemsdal TO, Sandvik L, Hjerkinn EM: **A randomized clinical trial on n-3 polyunsaturated fatty acids supplementation and all-cause mortality in elderly men at high cardiovascular risk.** *Eur J Cardiovasc Prev Rehabil* 2010, **17**:588–592.
13. Burr ML, Fehily AM, Gilbert JF, Rogers S, Holliday RM, Sweetnam PM, Elwood PC, Deadman NM: **Effects of changes in fat, fish, and fibre intakes on death and myocardial reinfarction: diet and reinfarction trial (DART).** *Lancet* 1989, **2**:757–761.
14. Hjerkinn EM, Seljeflot I, Ellingsen I, Berstad P, Hjermann I, Sandvik L, Arnesen H: **Influence of long-term intervention with dietary counseling, long-chain n-3 fatty acid supplements, or both on circulating markers of endothelial activation in men with long-standing hyperlipidemia.** *Am J Clin Nutr* 2005, **81**:583–589.
15. Mozaffarian D, Lemaitre RN, King IB, Song X, Huang H, Sacks FM, Rimm EB, Wang M, Siscovick DS: **Plasma phospholipid long-chain omega-3 fatty acids and total and cause-specific mortality in older adults: a cohort study.** *Ann Intern Med* 2013, **158**:515–525.
16. Galan P, Kesse-Guyot E, Czernichow S, Briancon S, Blacher J, Hercberg S: **Effects of B vitamins and omega 3 fatty acids on cardiovascular diseases: a randomised placebo controlled trial.** *BMJ* 2010, **341**:c6273.
17. Rauch B, Schiele R, Schneider S, Diller F, Victor N, Gohlke H, Gottwik M, Steinbeck G, Del Castillo U, Sack R, Worth H, Katus H, Spitzer W, Sabin G, Senges J: **OMEGA, a randomized, placebo-controlled trial to test the effect of highly purified omega-3 fatty acids on top of modern guideline-adjusted therapy after myocardial infarction.** *Circulation* 2010, **122**:2152–2159.
18. Kromhout D, Giltay EJ, Geleijnse JM: **n-3 fatty acids and cardiovascular events after myocardial infarction.** *N Engl J Med* 2010, **363**:2015–2026.
19. Hooper L, Thompson RL, Harrison RA, Summerbell CD, Ness AR, Moore HJ, Worthington HV, Durrington PN, Higgins JP, Capps NE, Riemersma RA, Ebrahim SB, Davey Smith G: **Risks and benefits of omega 3 fats for mortality, cardiovascular disease, and cancer: systematic review.** *BMJ* 2006, **332**:752–760.
20. Saravanan P, Davidson NC, Schmidt EB, Calder PC: **Cardiovascular effects of marine omega-3 fatty acids.** *Lancet* 2010, **376**:540–550.
21. Kromhout D, Yasuda S, Geleijnse JM, Shimokawa H: **Fish oil and omega-3 fatty acids in cardiovascular disease: do they really work?** *Eur Heart J* 2012, **33**:436–443.
22. DiNicolantonio JJ, O'Keefe JH, Lavie CJ: **The big ones that got away: omega-3 meta-analysis flawed by excluding the biggest fish oil trials.** *Arch Intern Med* 2012, **172**:1427–1428.
23. Rizos EC, Ntzani EE, Bika E, Kostapanos MS, Elisaf MS: **Association between omega-3 fatty acid supplementation and risk of major cardiovascular disease events: a systematic review and meta-analysis.** *JAMA* 2012, **308**:1024–1033.
24. Delgado-Lista J, Perez-Martinez P, Lopez-Miranda J, Perez-Jimenez F: **Long chain omega-3 fatty acids and cardiovascular disease: a systematic review.** *Br J Nutr* 2012, **107**(Suppl 2):S201–S213.
25. Adkins Y, Kelley DS: **Mechanisms underlying the cardioprotective effects of omega-3 polyunsaturated fatty acids.** *J Nutr Biochem* 2010, **21**:781–792.
26. Schmidt EB, Arnesen H, De Caterina R, Rasmussen LH, Kristensen SD: **Marine n-3 polyunsaturated fatty acids and coronary heart disease. Part I. Background, epidemiology, animal data, effects on risk factors and safety.** *Thromb Res* 2005, **115**:163–170.
27. **World medical association declaration of Helsinki: ethical principles for medical research involving human subjects.** *JAMA* 2013, **310**:2191–2194.
28. Mehran R, Rao SV, Bhatt DL, Gibson CM, Caixeta A, Eikelboom J, Kaul S, Wiviott SD, Menon V, Nikolsky E, Serebruany V, Valgimigli M, Vranckx P, Taggart D, Sabik JF, Cutlip DE, Krucoff MW, Ohman EM, Steg PG, White H: **Standardized bleeding definitions for cardiovascular clinical trials: a consensus report from the bleeding academic research consortium.** *Circulation* 2011, **123**:2736–2747.
29. Fox KA, Carruthers KF, Dunbar DR, Graham C, Manning JR, De RH, Buysschaert I, Lambrechts D, Van de Werf F: **Underestimated and under-recognized: the late consequences of acute coronary syndrome (GRACE UK-Belgian Study).** *Eur Heart J* 2010, **31**:2755–2764.

doi:10.1186/1471-2318-14-74
Cite this article as: Laake *et al.*: Effects of omega 3 supplementation in elderly patients with acute myocardial infarction: design of a prospective randomized placebo controlled study. *BMC Geriatrics* 2014 **14**:74.

Submit your next manuscript to BioMed Central and take full advantage of:

- **Convenient online submission**
- **Thorough peer review**
- **No space constraints or color figure charges**
- **Immediate publication on acceptance**
- **Inclusion in PubMed, CAS, Scopus and Google Scholar**
- **Research which is freely available for redistribution**

Submit your manuscript at
www.biomedcentral.com/submit

BioMed Central
