# Supplementary material for: Platelet-, monocyte-derived and tissue factor-carrying circulating microparticles are related to acute myocardial infarction severity
Source: PLoS One. 2017 Feb 16;12(2):e0172558. doi: 10.1371/journal.pone.0172558 (PMC5313202; doi:10.1371/journal.pone.0172558)
Supplement: S3 Appendix — (PDF) [file pone.0172558.s003.pdf]

Patient code	CIRCULATING MICROPARTICLES (AV+ cMP/µL plasma)					
	AV	CD142	CD61	CD142/CD61	CD62P	CD62L
1	180,67	3,33	116	1	1,67	10,33
2	403,67	4,33	379,67	0,67	7,33	8
3	126,5	5	94,5	3,5	1	4,5
4	1236,5	5	1272,5	1,5	39,5	147,5
5	758	12,33	948,33	3	17,67	7,33
6	198	2	156	0,5	2,5	28
7	487,5	2,5	363,5	1	5	17,5
8	560	1	494	0	7	5
9	102	1,5	97	1,5	1	1
10	297	1	245	0	3	9,5
11	219,5	1,5	245,5	1,5	4	4,5
12	676	8,5	654	0	24	40
13	442,5	1	401	1	5,5	16,5
14	477,5	0,5	451,5	0,5	7,5	6,5
15	1783,5	11,5	1689,5	0	31,5	18
16	670,5	3	552,5	0,5	8	18
17	292,5	1	331	0,5	14	11,5
18	449	2,5	475,5	0,5	7	6,5
19	277	0,5	261	0	1,5	11,5
20	1118,5	3	1036	0,5	14,5	31,5
21	725	4	670	0,5	14,5	8
22	346,5	3	384	0	4,5	7
23	214	1	211	0	8,5	8,5
24	609	2	589,5	0	6	11
25	224	3	125	2,5	4	21,5
26	184	3,5	89,5	0,5	5,5	25,5
27	1093	3,5	1746	0,5	22,5	16
28	306,5	1,5	234,5	0	2	7
29	937	3	826	0	4	9
30	900,5	3,5	623	1,5	18	7,5
31	705,5	1,5	667	0,5	17,5	2,5
32	140	0,5	122,5	0,5	8	8,5
33	169	1	128	0,5	1,5	22,5
34	547	12	383,5	1,5	25	21,5
35	127	2,5	88	1	2,5	13
36	393,5	3,5	325,5	1	5	10
37	320	7	215,5	1,5	12	6,5
38	217,5	1	257,5	1	5	6,5
39	89	5,5	59	2,5	0,5	4
40	87	1,5	61	0,5	3	5,5
41	201	1	175,5	0,5	6,5	4,5
42	137	6	142	3	6,5	5
43	214	3,5	209	1	3,5	10
44	1168	4	1186,5	0	24,5	13,5
45	466,5	8,5	315	2,5	8	22
46	790	12,5	539	2,5	69,5	31,5
47	189	2,5	145	0,5	2,5	7,5
48	377,5	3,5	300,5	0,5	12,5	23,5

Patient code	CIRCULATING MICROPARTICLES (AV+ cMP/µL plasma)					
	AV	CD142	CD61	CD142/CD61	CD62P	CD62L
49	505	5	384,5	0	8,5	34
50	293	1	214,5	0,5	10,5	5,5
51	739,5	2	637,5	0,5	23	9
52	105,5	1,5	47	0	2	4
53	527,5	3,5	474,5	0	15	6,5
54	252,5	2,5	251,5	0,5	9,5	10,5
55	154,5	2	156,5	0	7,5	5,5
56	21,67	0,42	17,08	0,42	0	3,33
57	128,5	1,5	114	1	6,5	7,5
58	190,5	2	199,5	0	12	3,5
59	498	12	549	8,5	38,5	8,5
60	594	1,5	530,5	0,5	18	9,5
61	546,5	4	568,5	2	7	14
62	342,5	1	412	1	7	10,5
63	2595,5	11,5	6224,5	1	122,5	43
64	822,5	6	940,5	3	19,5	7
65	363	8,5	401	4	9	25,5
66	329	3	287	0	5	12,5
67	505	2,5	416,5	1	6,5	13,5
68	652,5	17,5	431,5	1,5	65,5	34,5
69	205	6	173,5	2,5	0,5	9
70	137	0,5	110	0	0	2
71	613	2	753	0,5	28,5	20
72	681	4	676	0,5	15	11
73	611,5	59,5	642,5	45	15	10,5
74	420,5	4	359	0	7	17
75	1193	101	463,5	8	73	50,5
76	583	4,5	513	0	8,5	15,5
77	852,5	13	568,5	1	47	31
78	683	6	800	2,5	22,5	15,5
79	623,5	4,5	423	0,5	8,5	37
80	475	12,5	290,5	0,5	10,5	18
81	175,5	30,5	107,5	17,5	3,5	15,5
82	321,5	5,5	215	0,5	8	60,5
83	314	2	356,5	0	11	8,5
84	334,5	11,5	212,5	5	25,5	10
85	214,5	10,5	149,5	10	5	26
86	1697	58	1967	10	41,5	48,5
87	681	4	597,5	1,5	16	15
88	426,5	19,5	399	0,5	8,5	118
89	1342,5	54,5	782,5	3	94,5	45
90	1090	26	934	7,5	68,5	53
91	757	9	638	3	27	22,5
92	250,5	4	235,5	1	4	13
93	155,5	7	81	1,5	3,5	12,5
94	563,5	1,5	450	1	10	4,5
95	164,5	7	80,5	1	6	28
96	155,5	3,5	89	0,5	0,5	6,5

Patient code	CIRCULATING MICROPARTICLES (AV+ cMP/µL plasma)					
	AV	CD142	CD61	CD142/CD61	CD62P	CD62L
97	210,5	2,5	117,5	1	2	16
98	888,5	22	512	3,5	95,5	56
99	366,5	9,5	185	2,5	31	26
100	1157,5	44,5	520	5	119	125
101	1488,33	19,32	1330,32	2,76	55,89	46,23
102	543	9	444,5	3	23,5	17,5
103	212,5	2	105	0,5	4,5	15,5
104	1057,5	43,5	612	12	18,5	78
105	426,5	9	298	2,5	17	21,5
106	1283,5	27,5	260	3	51	60
107	1185	34,5	819,5	4	49,5	48
108	1316,5	7	779	0,5	11,5	8,5
109	857,5	10,5	705	1,5	20	33,5
110	881,5	29	619,5	13,5	10	31,5
111	966	38	612,5	7	38,5	61
112	398,5	6	269,5	1,5	4,5	18,5
113	844,5	22	789,5	6,5	21	32,5
114	803	27	609,5	6	24,5	54
115	763	19	572,5	10	24	31,5
116	506	33	269	2,5	15	34,5
117	519,5	21	329,5	4	9,5	21
118	714	30	447	3,5	26,5	24
119	1125	31	419	3,5	42	84,5
120	411,5	20	357,5	4,5	14,5	14
121	602	10,5	407,5	4	7,5	27
122	1060	52,5	1030,5	3,5	30,5	27,5
123	1318,5	37,5	1521,5	5,5	39,5	34,5
124	443	16,5	292,5	2	24,5	39
125	170	7,5	109	3	5,5	6
126	282	2	211	1	4	7,5
127	367	7,5	193	2	7,5	18,5
128	674,5	64	642,5	18	64	22
129	599,5	16,5	651	4	6	52,5
130	515	26,5	515	7,5	20	19
131	1034	39,5	1074	12,5	21	46,5
132	638,5	37	541,5	17	23	30,5
133	482	31	445,5	11	15,5	20,5
134	453	8	404,5	3	24,5	16,5
135	856	39	697	2,5	49	29
136	1757,5	78	1783	12,5	41,5	50,5
137	3576	157,5	2846	16	194	144
138	1072,5	42,5	738,5	3,5	42,5	48
139	748	21	745,5	6	35	26,5
140	610	12	428,5	3,5	14	28,5
141	805,5	18,5	499,5	4	14,5	63,5
142	423,5	9	382,5	1	6,5	28,5
143	792,5	11	688	2,5	14,5	46,5
144	1967,5	16,5	1924	3	56,5	42,5

Patient code	CIRCULATING MICROPARTICLES (AV+ cMP/µL plasma)					
	AV	CD142	CD61	CD142/CD61	CD62P	CD62L
145	908	13,5	796,5	2,5	17	13
146	480	17	372,5	5	17	16,5
147	638,5	21,5	468	1,5	20	23,5
148	2292,5	155	1804,5	8	98	47,5
149	996,5	13	849	4,5	25	34
150	531	9	520	2	41,5	20,5
151	734	10	652,5	2,5	33,5	18,5
152	1094,5	12,5	943	7	47,5	19
153	2444,5	15,5	2383	5,5	94,5	41
154	2648,5	21	2126	4,5	80,5	22
155	961	26	718	2	44	24
156	1189	32	708,5	4,5	66	41
157	2326,5	76	1727,5	7,5	136,5	69,5
158	630	159	305,5	5	54,5	26,5
159	1719	34,5	1330,5	6	79	36,5
160	567,5	6,5	470	4,5	34	25,5
161	581	9,5	450	6	40	22,5
162	3948	96	3656	5,5	204	78,5
163	1933,5	12,5	1780	2	124	26,5
164	1596,5	29,5	880	4,5	79	79,5
165	829	9,5	531,5	1	41,5	18,5
166	857	31,5	619	7	35	24
167	945,5	33	832,5	7	74,5	49
168	999,5	43	844	19,5	76	52
169	1028	71	824,5	5	52,5	41,5
170	1410	35	623	3	61,5	85,5
171	507,5	14,5	165,5	1	30	35
172	704	17,5	647	6,5	27	30,5
173	425	42	326,5	20	30,5	29,5
174	1718	26	1202	9,5	150	81
1106	727	8	640	4,5	45,5	13
1181	846	8	721,5	1,5	42,5	30
1182	2192,5	101	1697,5	54	168,5	97
1183	1235,5	15	997,5	4,5	69	32,5
1184	1040	15,5	570	2,5	76	32,5
1185	568,5	21,5	510,5	14	18	12
1186	449	8,5	322,5	1,5	19	21
1187	672	20,5	445,5	5	32,5	16,5
1188	705	30	519	11	51	16
1189	667,5	10	393,5	2	28	18,5
1190	1297,5	18,5	502,5	5	57	43,5
1191	1179	41	1238,5	6,5	142,5	53,5
1192	933,5	19,5	837	5,5	55	53
1193	2149	14,5	1888,5	5	87,5	37,5
1194	1759,5	9,5	1384,5	3	64	27,5
1195	1897,5	30	1415,5	5	135	43
1196	1922,5	57,5	1033,5	13,5	135	118,5
1197	1067	16	700	2,5	74	42,5

Patient code	CIRCULATING MICROPARTICLES (AV+ cMP/µL plasma)					
	AV	CD142	CD61	CD142/CD61	CD62P	CD62L
1198	1186,5	22,5	974	3,5	74,5	48
1199	822	32,5	502,5	17	31,5	17,5
1200	419,5	4	224,5	1	10,5	6,5
1201	433	35	345	11	32	28
1202	744,5	51	469	3,5	69	41
1203	1570	74	1512	9,5	84	61
1204	1469	58,5	1126	4,5	54	50,5
1205	1148	53	927	2	47	42

Patient code	CIRCULATING MICROPARTICLES (AV+ cMP/µL plasma)					
	CD146	CD62E	CD146/CD62E	CD309	CD31	CD42b
1	2,33	22,33	1	6,67	27,33	47,67
2	0,67	14,67	0,67	6,33	188,67	216,33
3	0,5	9	0,5	6,5	24,5	32
4	2,5	31,5	0	12,5	620,5	303,5
5	3,33	18	0,33	135,67	291,67	309,67
6	1,5	35,5	0,5	17	29	86
7	1,5	54	0	17,5	168,5	294
8	2	9,5	0,5	6,5	143	280,5
9	0,5	2	0,5	2,5	42,5	43,5
10	0,5	11	0,5	2	88,5	84
11	2	15,5	0	2,5	104	154,5
12	2,5	51	0,5	18	289,5	438,5
13	1	25,5	0	13	241,5	197
14	1	20,5	0	9	243,5	216,5
15	6,5	57	1,5	52	1075	1376
16	1	33	0	12	291	259
17	1,5	21,5	0,5	5,5	84,5	111
18	0,5	11,5	0	8	301	259,5
19	0,5	20	0	9	115,5	133,5
20	1,5	80	0,5	42,5	559	598,5
21	1	16	1	12,5	333	277,5
22	1	10	0	3,5	153	250
23	0,5	15	0	4	83,5	135
24	0,5	14	0	7	258	329
25	2,5	45,5	0	26	43,5	83
26	1,5	59,5	0,5	40,5	44,5	82
27	2	20,5	0,5	16,5	639	845,5
28	0	12	0	7,5	145,5	158
29	1,5	29,5	0	13,5	510,5	460,5
30	1,5	16	0	10	414,5	191
31	2	12	0	7,5	355	231,5
32	0,5	16	0	7	56	91
33	0	38	0	10	35,5	76,5
34	2	68,5	1,5	47	234,5	187
35	0,5	40	0	16,5	28,5	51,5
36	1	27	0	12	102	148,5
37	1	18	0	8	110,5	168,5
38	1,5	17,5	0,5	6,5	81	127
39	0,5	12,5	0,5	5,5	19,5	20,5
40	1	13,5	0,5	7,5	23,5	34
41	0	12	0	3,5	85,5	88
42	0,5	12,5	0	1	40	61
43	1	21,5	0	16	80	119,5
44	1,5	24	0	10,5	456,5	804,5
45	0	22,5	0	16,5	136	137
46	2,5	104	2,5	86	391	313,5
47	1	8	0,5	4,5	127,5	103,5
48	0,5	49,5	0	35,5	120	131,5

Patient code	CIRCULATING MICROPARTICLES (AV+ cMP/µL plasma)					
	CD146	CD62E	CD146/CD62E	CD309	CD31	CD42b
49	3	85,5	1,5	29	149	266
50	0,5	4,5	0	4	67,5	118
51	1	14,5	0,5	9	286,5	370,5
52	0	2,5	0	3	25	20,5
53	1	12,5	0	8,5	208	249
54	2,5	27	1	9,5	89	158,5
55	1,5	5	0	3,5	61	81
56	0,42	12,08	0,83	2,92	5,42	10,83
57	1	4,5	0	2,5	30	37
58	1	10,5	0	4	74	76,5
59	1	17,5	0,5	5	294	397,5
60	0,5	9,5	0	5,5	314	444
61	1,5	14	0,5	2	210,5	279,5
62	0,5	10	0	3	139,5	125
63	4,5	46,5	0,5	69,5	1708,5	912
64	1	47,5	1	6	378	380
65	2	27	0,5	6,5	146,5	195,5
66	1,5	14	0	8,5	102,5	145,5
67	0,5	22,5	0	8,5	168	135
68	2,5	123,5	0,5	81	219,5	187,5
69	0	15	0	6,5	86	46
70	0,5	33	0,5	8,5	33	31,5
71	1,5	42	1	13	405	459,5
72	1	31	0	13	357	438
73	1	65	0	9,5	341	305,5
74	3	49	1	16	119	141,5
75	12	151	4,5	145,5	196	254
76	3	21	0	8,5	272,5	197
77	3	71,5	0	61,5	312,5	172
78	1	23	0	11,5	291,5	512
79	1,5	80	0,5	34,5	243,5	303,5
80	1	22,5	0	11	142	122
81	0	45,5	0	16,5	43	58
82	2	85	2	36	95	80,5
83	0,5	10,5	0	4	137	232,5
84	0	37	0	26,5	105	67
85	1	48,5	1,5	21,5	109,5	135,5
86	10,5	124,5	2,5	204	862	974,5
87	1	15	0,5	11	319,5	317,5
88	2	100,5	1,5	56,5	145	141,5
89	12	159	3	182	351	353
90	4,5	194	3,5	192,5	467	685,5
91	1	57,5	1	50	459	532,5
92	0,5	19	0	4	113,5	172
93	0,5	36	0,5	20	73	81,5
94	0,5	13	0	7,5	258,5	200,5
95	0,5	29	1	23,5	49	54
96	0,5	12,5	0,5	22	23,5	16

Patient code	CIRCULATING MICROPARTICLES (AV+ cMP/µL plasma)					
	CD146	CD62E	CD146/CD62E	CD309	CD31	CD42b
97	0,5	19,5	0,5	17,5	65,5	40,5
98	4,5	233,5	2,5	175	379	379,5
99	0,5	72,5	1,5	63,5	113	96,5
100	6	377	6	363	545	566,5
101	6,21	146,97	2,07	91,77	954,96	1148,16
102	0,5	48	0,5	38	254	344
103	0	48	1	44	55	74,5
104	3	218,5	0,5	156,5	195	310
105	2,5	69,5	1	47,5	112,5	154
106	7,5	336,5	6	263	279,5	430
107	3,5	294	3	114	340,5	588
108	3	44	0,5	34,5	627,5	387
109	2,5	94	0,5	79,5	440	552,5
110	7	96	1,5	94,5	410	316
111	7,5	132,5	3,5	163	216	198
112	2	54	0,5	46,5	126	137
113	9	69,5	3	55	286	413
114	4,5	99	2,5	89,5	218,5	276
115	3	55,5	0,5	81,5	168,5	162,5
116	3	96,5	2	53	171,5	147,5
117	4,5	47	2	67	140	140
118	5	121	1,5	60	129,5	204,5
119	11	194,5	6,5	129	165,5	249,5
120	2	35,5	0,5	55,5	175,5	104
121	6,5	89	1,5	58	171	134,5
122	4	76,5	1	70,5	478,5	339
123	4,5	76,5	1	105	950	545,5
124	3	116	0,5	56,5	192,5	259
125	1,5	16	1	9	67,5	66
126	1	23,5	0	13	129	100,5
127	0,5	80,5	0,5	63,5	100	123
128	8	66	3	67	250,5	173,5
129	3	164,5	3	92,5	248,5	304
130	4,5	62,5	1,5	44,5	246,5	287,5
131	8	82	1,5	127	477,5	458,5
132	5	54	0,5	117,5	104,5	70
133	1,5	40	0	65,5	172	101
134	3	50	1,5	76	121	76,5
135	6	47	0	115	260,5	301
136	10	139	1,5	211	890	781,5
137	17	211,5	4,5	469,5	1006,5	779
138	7	78	1,5	100	448,5	261
139	5	63,5	1,5	114,5	286,5	180,5
140	2,5	70,5	1	38,5	344,5	358,5
141	2	122	1	109	368,5	359,5
142	1	108	1,5	79,5	227	152,5
143	3,5	100,5	2,5	224	399,5	212
144	4,5	117,5	2,5	162,5	1274	782

Patient code	CIRCULATING MICROPARTICLES (AV+ cMP/µL plasma)					
	CD146	CD62E	CD146/CD62E	CD309	CD31	CD42b
145	2,5	63,5	0	44	439	526,5
146	5,5	63,5	0,5	53	204,5	198
147	2,5	71	0,5	98,5	201	152
148	15,5	195	2	313,5	1232	741
149	7	107,5	2,5	133,5	518	248
150	2	72,5	1	48,5	325,5	333
151	2,5	64,5	0,5	53,5	599,5	580,5
152	5,5	44,5	1	36,5	750,5	509,5
153	5	116,5	0	73	1807	644
154	7,5	86	1,5	97,5	1983	954
155	8	102,5	2,5	107	436,5	521,5
156	12,5	127,5	3,5	136	550,5	505
157	10,5	173	5	232	1139	749,5
158	7,5	105,5	2,5	104,5	171,5	135,5
159	8	82,5	1,5	185,5	927	579,5
160	2	83,5	1,5	47	264	281,5
161	2	83	2,5	50,5	209	242
162	10	99	1,5	89,5	3119	1810
163	4,5	81	0,5	57,5	1243	1381,5
164	6	252	4	174,5	577,5	382
165	2,5	90	2,5	64,5	366	256,5
166	5	62	0,5	87,5	383	401,5
167	9	132	2	134,5	419,5	422,5
168	8,5	132	5	163	326	252,5
169	6	87,5	1	137	472,5	210,5
170	6	338	5	220,5	460,5	626
171	2	80,5	2	62	140	139,5
172	4	69,5	1,5	76	190,5	181,5
173	2,5	90	2,5	46	200	239
174	6	346	3,5	318	1007,5	954,5
1106	2,5	20,5	0,5	14,5	142	84,5
1181	3,5	72	1,5	108,5	434,5	359
1182	10	283,5	6,5	246	1368,5	944
1183	6,5	71,5	1,5	134,5	716	379,5
1184	4,5	159,5	2,5	158,5	421,5	360
1185	1	47,5	1	30	349	212,5
1186	0,5	12,5	0	35,5	205,5	133
1187	3	75,5	1	57,5	331,5	174
1188	1	80,5	0,5	64,5	365,5	192
1189	2	81,5	1	64,5	328	263
1190	7	227,5	5,5	160	394,5	347
1191	6,5	69,5	0,5	120,5	501,5	635
1192	7	71	1,5	107,5	500,5	607
1193	4,5	102,5	0,5	103,5	1528	1473,5
1194	5,5	108	1,5	74,5	956	1010
1195	4,5	193	2	162	1046	682
1196	8	476	9,5	383,5	733,5	735
1197	2,5	96,5	1,5	50,5	518,5	268,5

Patient code	CIRCULATING MICROPARTICLES (AV+ cMP/µL plasma)					
	CD146	CD62E	CD146/CD62E	CD309	CD31	CD42b
1198	5,5	112	2	86,5	674,5	321
1199	3	109,5	1,5	90,5	387,5	251
1200	1	23	1	25,5	170	91,5
1201	4	51	1,5	94,5	125	82,5
1202	5	97,5	1,5	147	235	163
1203	10,5	129,5	3,5	219	551	194,5
1204	9	138,5	3	179	389,5	290
1205	8	115	1	136	365	182

Patient code	CIRCULATING MICROPARTICLES (AV+ cMP/µL plasma) CD31/CD42b	CD31+/CD42b-	CD235ab	CD3/CD45	SMC	CD142/SMC
1	20,33	7	15,33	4,67	2,33	0,67
2	129,67	59	10,33	2,67	3	1,33
3	16,5	8	7,5	3	1,5	1,5
4	201,5	419	9	2,5	3,5	1
5	208	83,67	8	93,33	1	0,33
6	28	1	53	9	10,5	1
7	154	14,5	49,5	14,5	3,5	0
8	112,5	30,5	17	2,5	3,5	0,5
9	25	17,5	0,5	0,5	0,5	0,5
10	51,5	37	11,5	1	3	1,5
11	104	0	4	3,5	2,5	0,5
12	281	8,5	31	11	6,5	2
13	149,5	92	26	10,5	5,5	2
14	160,5	83	17	4,5	3,5	1,5
15	1116	0	24	7	5	2,5
16	166,5	124,5	46	2,5	8	2
17	86,5	0	0,5	1	4,5	4
18	222,5	78,5	4	0	4	2
19	76	39,5	15	4	1	0
20	407	152	72	19,5	14,5	3
21	192,5	140,5	36	2,5	3	2,5
22	156,5	0	8,5	5	6	4,5
23	80	3,5	17,5	4	2	1,5
24	193	65	11	2,5	4,5	1
25	35	8,5	38	21	12	4,5
26	41,5	3	68	3,5	15	3,5
27	660	0	4,5	2	7,5	3,5
28	119	26,5	12	3	5	2,5
29	347	163,5	15,5	6,5	6,5	3,5
30	139,5	275	2,5	3	2	1
31	185,5	169,5	3,5	1	3	2
32	57,5	0	7,5	1,5	3	0,5
33	30	5,5	39,5	14	16,5	5,5
34	149,5	85	64	21,5	0,5	0
35	27	1,5	35,5	11,5	11	4,5
36	80,5	21,5	24,5	7	9,5	4,5
37	99,5	11	10,5	5	6	3
38	83	0	7	2	3,5	2,5
39	13	6,5	8,5	7,5	6,5	4,5
40	21,5	2	7,5	3,5	6	4
41	66,5	19	6,5	3,5	3	1
42	37	3	9	1,5	14	13,5
43	79,5	0,5	26	10	3	1,5
44	443,5	13	20,5	3,5	9,5	4,5
45	91,5	44,5	34	11	4,5	1,5
46	251,5	139,5	103,5	36,5	0	0
47	74	53,5	15	3	1,5	0
48	81	39	47,5	30,5	7,5	2,5

Patient code	CIRCULATING MICROPARTICLES (AV+ cMP/µL plasma)					
	CD31/CD42b	CD31+/CD42b-	CD235ab	CD3/CD45	SMC	CD142/SMC
49	161,5	0	55	17	5,5	2
50	69	0	11,5	4	4	2,5
51	253	33,5	10	5	4,5	1,5
52	13,5	11,5	7,5	1,5	2	0,5
53	157	51	4,5	0,5	3	2
54	95	0	14	4	3,5	2
55	58,5	2,5	6,5	3,5	2,5	1
56	4,58	0,83	7,92	3,75	3,75	3,33
57	23,5	6,5	4,5	1	2	1,5
58	58	16	16	2	2,5	0,5
59	310	0	3	6	4	1,5
60	315	0	9	2,5	2,5	1,5
61	165,5	45	16	5	0,5	0
62	94,5	45	90,5	3	13	3
63	838	870,5	15	7	0	0
64	253,5	124,5	3	1,5	1	0,5
65	139,5	7	20,5	11,5	7	1
66	88	14,5	15,5	6,5	9,5	2
67	93,5	74,5	32	10,5	8,5	3
68	128	91,5	121,5	38,5	0	0
69	36	50	7	13	4	0,5
70	16	17	27,5	3,5	4	1,5
71	405,5	0	21	12	6	2
72	331,5	25,5	11,5	7	7	2
73	244,5	96,5	39,5	30	3,5	0,5
74	81,5	37,5	52	18,5	5	1
75	137,5	58,5	80	14	0	0
76	155,5	117	9	2,5	1,5	0,5
77	121,5	191	54	14	0	0
78	314,5	0	27	12,5	17,5	5
79	211	32,5	93	33,5	12	3
80	91,5	50,5	25,5	7	13,5	5
81	37,5	5,5	21	29,5	7,5	1
82	65,5	29,5	45	4	14	1,5
83	144,5	0	6	2	0,5	0
84	45	60	30,5	18	0	0
85	100	9,5	34	36	11	5
86	750	112	35,5	21	0	0
87	239	80,5	9	6,5	4	2,5
88	85,5	59,5	103	53	14,5	2
89	252	99	349,5	21,5	0	0
90	480,5	0	151,5	73	19,5	2,5
91	432,5	26,5	43	18,5	1,5	0,5
92	116	0	13	4,5	2,5	0
93	57	16	20,5	14,5	1,5	0,5
94	149	109,5	6,5	5	5	2,5
95	29	20	43,5	15	9	3
96	7,5	16	8,5	2	2,5	1,5

	CIRCULATING MICROPARTICLES (AV+ cMP/µL plasma)					
**Patient code**	**CD31/CD42b**	**CD31+/CD42b-**	**CD235ab**	**CD3/CD45**	**SMC**	**CD142/SMC**
97	27	38,5	19	3,5	4,5	3,5
98	258,5	120,5	217	69	17	2
99	68	45	65,5	22	6	1
100	422	123	392	98	28	6
101	962,55	0	126,96	30,36	17,94	6,9
102	243,5	10,5	53,5	17,5	7	2
103	45	10	48	15,5	1,5	1
104	163	32	327,5	70,5	22	4
105	86,5	26	70,5	13	11	4
106	215,5	64	772	57	0	0
107	345	0	350,5	85	60	5,5
108	318	309,5	33,5	34	0	0
109	386,5	53,5	66,5	37	16	2
110	224,5	185,5	74,5	63,5	16,5	3,5
111	100,5	115,5	257	21,5	20,5	3,5
112	82,5	43,5	69,5	14	8	3,5
113	269	17	70	12,5	3	1
114	177,5	41	141	30	18,5	1
115	85	83,5	33	13	5	0,5
116	99	72,5	112	20	16	3
117	71,5	68,5	154	17,5	6	0,5
118	104,5	25	173	28,5	8,5	1
119	112	53,5	459	42	25	1
120	70,5	105	31	23,5	0	0
121	78	93	113	23	12,5	2,5
122	279,5	199	53,5	16	2	0
123	407	543	51	12,5	7,5	3
124	192,5	0	123	41	29,5	3
125	50	17,5	13,5	8,5	16,5	0
126	79,5	49,5	13,5	5	2	1
127	67	33	89,5	19	8	1
128	147,5	103	60	22,5	0	0
129	190,5	58	147	26,5	12	1,5
130	231	15,5	25	26,5	6,5	0,5
131	343,5	134	53	13	10,5	2,5
132	44,5	60	86	38	9,5	3
133	68,5	103,5	40	12,5	8,5	3,5
134	47	74	51	15	13	11
135	248	12,5	48,5	20	8,5	1,5
136	627	263	54,5	30	29	8,5
137	576	430,5	785,5	40,5	12	1
138	213,5	235	59,5	32	13	2,5
139	133,5	153	58	19,5	10,5	1
140	303	41,5	68,5	22	8	2,5
141	265	103,5	184	51,5	11	2
142	106,5	120,5	113	31,5	4	1
143	169,5	230	71	18,5	5	1
144	675	599	136	15,5	10	1,5

Patient code	CIRCULATING MICROPARTICLES (AV+ cMP/µL plasma)					
	CD31/CD42b	CD31+/CD42b-	CD235ab	CD3/CD45	SMC	CD142/SMC
145	430,5	8,5	29	17	6	2,5
146	144	60,5	52,5	16,5	3,5	1
147	97	104	362,5	13,5	8,5	2,5
148	650	582	38	35,5	14,5	3,5
149	202	316	49,5	29	24,5	9,5
150	306,5	19	29,5	25	15	3
151	509	90,5	37	20,5	8	4
152	456,5	294	27,5	10	12,5	5
153	607,5	1199,5	37,5	19,5	10	1
154	830	1153	34	22,5	22	4,5
155	386	50,5	79,5	13	5	0,5
156	368,5	182	77	36	2	0
157	573,5	565,5	148	52	13,5	2,5
158	77,5	94	152,5	27,5	6	2
159	393,5	533,5	20	26,5	5	2
160	233	31	56,5	34,5	4	0,5
161	155	54	61	35	9	2
162	1680	1439	60	16,5	6,5	3,5
163	1251	0	44,5	14,5	22	3
164	257	320,5	313,5	62,5	18	3,5
165	192	174	110	20,5	9	3
166	355,5	27,5	48,5	16	15,5	5
167	299,5	120	167,5	41	18	2
168	147,5	178,5	204,5	33	19	2
169	163,5	309	57,5	14,5	9	2
170	405	55,5	585,5	88	0	0
171	101,5	38,5	81	37,5	10	2
172	104,5	86	54	33,5	9,5	3,5
173	192	8	93	48	8	2
174	727	280,5	340	96	31,5	3,5
1106	60,5	81,5	96	2	28,5	3,5
1181	307,5	127	69	19	7,5	1,5
1182	825,5	543	247,5	102,5	17,5	7
1183	338,5	377,5	46,5	15	1,5	0,5
1184	288,5	133	134	29	13,5	3
1185	186,5	162,5	24,5	22	2	0,5
1186	104,5	101	30	40	6	2,5
1187	132	199,5	52	30,5	5	1
1188	149	216,5	76	26	8,5	4
1189	210	118	78	24	9	2,5
1190	243,5	151	530,5	28	12,5	4,5
1191	508,5	0	15,5	11	2,5	1
1192	469,5	31	52	21,5	5,5	2
1193	1288,5	239,5	55	20,5	4,5	0,5
1194	719,5	236,5	92,5	22	2,5	1,5
1195	607,5	438,5	151,5	64,5	15	0,5
1196	516	217,5	493	181,5	18	2,5
1197	223	295,5	63,5	36,5	4,5	0,5

	CIRCULATING MICROPARTICLES (AV+ cMP/µL plasma)					
**Patient code**	**CD31/CD42b**	**CD31+/CD42b-**	**CD235ab**	**CD3/CD45**	**SMC**	**CD142/SMC**
1198	275	399,5	96,5	43,5	4,5	1,5
1199	195,5	192	88	23,5	6	0
1200	71	99	25,5	6,5	0,5	0,5
1201	48,5	76,5	68	24,5	8	2
1202	106,5	128,5	173,5	38,5	8	2
1203	159	392	66	18,5	8	2
1204	182	207,5	144	26	8	2
1205	138	227	116	28	8	2

	CIRCULATING MICROPARTICLES (AV+ cMP/µL plasma)					
**Patient code**	**CD45**	**CD11b**	**CD14**	**CD11b/CD14**	**CD142/CD14**	**CD45+/CD3-/CD14-**
1	67,67	4,33	1,33	0	0	61,67
2	53	2	3,67	0,33	1,33	46,67
3	52,5	2,5	2,5	1	0	47
4	53,5	3	4,5	0,5	1	46,5
5	176,67	22,33	27,33	19,33	0,33	56
6	40	7,5	2	0	0	29
7	55,5	5	2	0,5	0	39
8	18	1	3	0	0	12,5
9	31	0,5	1,5	0	0	29
10	66,5	1	1	0	0	64,5
11	49	1	1	0	0	44,5
12	96,5	3	3,5	1	0	82
13	87	2	1,5	0	0	75
14	36,5	2,5	0,5	0	0	31,5
15	110	9	3	0	0	100
16	74,5	3,5	3	0	0	69
17	35,5	1	2	0	0	32,5
18	67,5	0,5	1	0	0	66,5
19	42	1,5	2	0	0	36
20	105,5	3,5	3,5	0,5	0	82,5
21	85,5	2	1,5	0	0	81,5
22	78	4	3	0,5	0,5	70
23	20,5	0,5	1	0	0	15,5
24	74	2,5	2,5	0	1	69
25	112	6	2	1	0,5	89
26	48	2	2,5	0	0	42
27	30	5	2	0,5	0	26
28	68,5	3,5	1	0	0	64,5
29	91,5	0	0	0	0	85
30	112	6	4	0,5	0,5	105
31	38,5	0,5	2	0,5	0	35,5
32	40,5	1	2	0,5	0	37
33	58,5	3,5	2	0	0	42,5
34	145,5	12,5	4	1	0	120
35	51,5	1,5	1,5	0,5	0	38,5
36	77	3	2	0	0	68
37	61,5	1	2	0	1,5	54,5
38	46,5	3	1	0	0	43,5
39	45,5	3	3	2	1	35
40	19	2	1,5	0	0	14
41	21,5	1	1	0	0	17
42	13	1,5	8	1	4,5	3,5
43	71	7	2	0,5	0	59
44	60,5	3	6,5	0	0	50,5
45	121	9	1,5	0	0,5	108,5
46	182,5	9,5	5	0	1	141
47	35	2,5	2,5	0	0	29,5
48	93	5,5	3	0	0,5	59,5

	CIRCULATING MICROPARTICLES (AV+ cMP/µL plasma)					
**Patient code**	**CD45**	**CD11b**	**CD14**	**CD11b/CD14**	**CD142/CD14**	**CD45+/CD3-/CD14-**
49	49,5	10	2	0	0,5	30,5
50	40,5	1,5	1	0	0	35,5
51	57	1,5	4	0	0	48
52	30,5	2	0	0	0	29
53	40	2	4,5	0	0	35
54	51,5	4	1,5	0	0,5	46
55	26,5	2,5	2	0,5	0	21
56	7,5	1,67	1,25	0	0	2,5
57	36	1,5	1	0	0	34
58	34,5	1	1	0	0	31,5
59	40,5	7	6	3,5	5	28,5
60	58	1,5	3,5	0	0	52
61	56,5	1	1,5	0	1,5	50
62	36,5	0,5	1	0	0	32,5
63	130	16	6,5	0,5	0,5	116,5
64	59	1,5	9	0,5	1	48,5
65	169,5	7	5	2	1	153
66	96,5	2	3	0	0,5	87
67	103	4	3,5	0	0,5	89
68	119,5	8,5	7,5	0	1	73,5
69	137,5	0,5	1	0	0	123,5
70	50,5	1,5	1	0	0	46
71	80	3,5	1,5	0	0	66,5
72	80,5	6	3,5	0	1	70
73	61	25	33	24,5	30,5	0
74	75,5	4,5	4	0,5	0,5	53
75	97,5	52,5	18,5	0,5	4	65
76	150,5	2,5	3,5	0	0	144,5
77	170,5	8	6	1	0,5	150,5
78	60,5	11,5	3,5	0	1	44,5
79	152	8	3,5	1	0,5	115
80	100,5	4,5	6	0	2	87,5
81	46	5,5	31	4	24,5	0
82	65	8	4	0,5	0	57
83	40,5	3,5	1,5	0	0	37
84	118	18,5	15,5	10,5	5	84,5
85	94,5	14	11,5	5,5	7,5	47
86	216,5	25,5	19,5	0,5	1,5	176
87	124,5	5,5	5	1	0	113
88	118,5	9,5	11,5	1	3	54
89	140	38,5	15,5	1	1	103
90	202	23	10	1,5	1	119
91	151,5	6,5	10	0,5	0	123
92	44	4,5	1	0	0	38,5
93	49,5	5,5	7	3,5	5	28
94	99,5	3,5	1	0	0	93,5
95	28	4,5	2	0	0,5	11
96	51,5	1,5	2	0,5	0,5	47,5

Patient code	CIRCULATING MICROPARTICLES (AV+ cMP/µL plasma)					
	CD45	CD11b	CD14	CD11b/CD14	CD142/CD14	CD45+/CD3-/CD14-
97	77	3	4	0,5	0	69,5
98	228	18	9	1,5	0	150
99	167	19,5	3	0,5	1	142
100	438	45,5	15	4	1	325
101	135,93	13,11	8,28	2,07	0	97,29
102	107,5	5,5	3,5	1,5	0,5	86,5
103	94	6	2,5	0	0	76
104	187	21	9	1,5	2	107,5
105	160	6,5	4,5	0,5	1	142,5
106	298,5	25	17	2	1	224,5
107	214,5	28	11	3	2	118,5
108	356,5	28,5	4	1,5	1	318,5
109	243	9,5	4	0,5	0,5	202
110	267,5	19,5	34	8,5	9,5	170
111	136	22,5	8,5	0,5	0,5	106
112	141	17	5,5	0,5	0,5	121,5
113	172	23,5	6	1	0	153,5
114	229,5	23	13	1,5	3	186,5
115	250	24	9	1,5	0,5	228
116	163	17,5	11	1,5	3	132
117	133,5	14,5	7	0,5	2	109
118	153	27,5	10,5	2	2	114
119	196,5	24	10,5	1	2,5	144
120	209	12,5	5,5	1,5	0	180
121	210	19,5	6	0,5	0	181
122	180	11,5	17	0,5	1,5	147
123	128,5	17,5	8	0	0,5	108
124	146	8	4	0	1	101
125	91	3	5	2,5	2,5	77,5
126	128	5	1	0,5	0	122
127	146	8,5	1,5	0,5	0,5	125,5
128	141,5	14,5	19,5	1	5,5	99,5
129	182,5	13	6	1	0,5	150
130	184	25,5	2,5	4	1,5	155
131	202	42,5	10,5	1	1	178,5
132	162	25,5	33	10	14,5	91
133	106,5	20,5	7,5	2	0,5	86,5
134	172,5	24	11	1	0,5	146,5
135	159,5	38,5	11,5	1,5	3	128
136	223	62	16,5	1,5	3	176,5
137	327	65,5	37,5	3,5	5,5	249
138	308	48,5	11	0,5	2,5	265
139	149	43,5	14	1,5	2	115,5
140	214,5	23,5	3,5	2,5	1	189
141	370,5	24,5	9	4	1	310
142	188,5	8	3,5	1	1	153,5
143	210	6,5	5	1	0	186,5
144	168,5	13,5	10,5	0,5	1	142,5

	CIRCULATING MICROPARTICLES (AV+ cMP/µL plasma)					
**Patient code**	**CD45**	**CD11b**	**CD14**	**CD11b/CD14**	**CD142/CD14**	**CD45+/CD3-/CD14-**
145	205,5	15	4	0	0,5	184,5
146	179,5	11	5	1	0	158
147	167	15	6,5	0,5	1,5	147
148	313,5	44	23,5	1,5	3	254,5
149	278,5	23	9	2,5	0,5	240,5
150	198,5	10	3,5	0	0	170
151	200,5	5,5	3,5	0,5	0	176,5
152	123,5	9	5,5	0,5	0	108
153	202,5	15,5	6	0	0	177
154	392,5	21,5	14,5	1,5	2	355,5
155	99,5	24,5	9	1,5	1	77,5
156	267	34	13	1	2	218
157	398,5	55,5	18	1	1,5	328,5
158	101	19	85,5	1	73,5	0
159	240,5	102,5	28,5	3,5	1	185,5
160	229,5	12,5	3	1	0,5	192
161	175	5,5	5,5	0,5	0,5	134,5
162	120	18,5	47	3	25	56,5
163	96,5	9,5	5,5	0,5	0	76,5
164	192,5	20	11	0	4	119
165	132,5	8	8	0,5	0	104
166	174,5	23	6,5	1,5	1	152
167	273,5	29	10	0,5	0,5	222,5
168	272	55	13	3	2	226
169	140	23,5	12,5	0	1,5	113
170	156,5	29,5	11,5	2	2	57
171	190	20	8	1	0,5	144,5
172	234,5	13,5	13,5	0,5	4,5	187,5
173	119	15,5	19	9	18	52
174	346	36,5	18,5	5,5	1	231,5
1106	5,5	90	4,5	1,5	0	60
1181	211	5,5	7	0	0	185
1182	267,5	67,5	92	50	59	73
1183	140,5	14	5	0	1	120,5
1184	232,5	13	13	1	0	190,5
1185	72,5	10	17,5	4,5	16,5	33
1186	239,5	21,5	2	0,5	0,5	197,5
1187	233	15,5	6	2	1	196,5
1188	107	20	26	9	15	55
1189	189,5	12,5	5	0	0	160,5
1190	167	8	8,5	0	1	130,5
1191	104,5	31	9,5	2,5	1	84
1192	149	10	4,5	0	1	123
1193	269,5	15	6,5	1,5	0,5	242,5
1194	177,5	16	6,5	0	1,5	149
1195	324	26	15,5	1	4	244
1196	542	36	20,5	0,5	4,5	340
1197	298,5	14	8	0,5	1	254

	CIRCULATING MICROPARTICLES (AV+ cMP/µL plasma)					
**Patient code**	**CD45**	**CD11b**	**CD14**	**CD11b/CD14**	**CD142/CD14**	**CD45+/CD3-/CD14-**
1198	214,5	10,5	8,5	0	1	162,5
1199	265	15,5	28,5	4	11	213
1200	76	8,5	2	0,5	0,5	67,5
1201	223	11,5	9	0,5	0	189,5
1202	263,5	38	14,5	1	1,5	210,5
1203	187	66,5	24	3	1,5	144,5
1204	173	14	17,5	1	0,5	129,5
1205	237	46	13	0	0	196
